# Synthesis and Molecular Modeling of Antioxidant and Anti-Inflammatory Five-Membered Heterocycle–Cinnamic Acid Hybrids

**DOI:** 10.3390/molecules30153148

**Published:** 2025-07-27

**Authors:** Konstantinos Theodoridis, Eleftherios Charissopoulos, Dimitra Tsioumela, Eleni Pontiki

**Affiliations:** Laboratory of Pharmaceutical Chemistry, Faculty of Health Sciences, School of Pharmacy, Aristotle University of Thessaloniki, 54124 Thessaloniki, Greece; kontheodoridis@hotmail.com (K.T.); echariss@pharm.auth.gr (E.C.); detsioum@gmail.com (D.T.)

**Keywords:** cinnamic acid derivatives, 1,2,4-triazole, 1,3,4-oxadiazole, chimeric molecules, anti-inflammatory, antioxidant

## Abstract

In this study, the design and synthesis of a novel series of cinnamic acid and 1,2,4-triazole hybrids were reported, aiming to enhance antioxidant and lipoxygenase inhibitory activities through pharmacophore combination. Cinnamic acid derivatives and 1,2,4-triazoles exhibit a broad spectrum of biological activities; therefore, by synthesizing hybrid molecules, we would like to exploit the beneficial characteristics of each scaffold. The general synthetic procedure comprises three synthetic steps, starting from the reaction of appropriate substituted cinnamic acid with hydrazine monohydrate in acetonitrile with cyclohexane and resulting in the formation of hydrazides. Consequently, the hydrazides reacted with phenylisothiocyanate under microwave irradiation conditions. Then, cyclization proceeded to the 1,2,4-triazole after the addition of NaOH solution and microwave irradiation. All the synthesized derivatives have been studied for their ability (a) to interact with the free radical DPPH, (b) inhibit lipid peroxidation induced by AAPH, and (c) inhibit soybean lipoxygenase. The synthesized derivatives have shown significant antioxidant activity and have been proved to be very good lipoxygenase inhibitors. Compounds **4b** and **4g** (IC_50_ = 4.5 μM) are the most potent within the series followed by compound **6a** (IC_50_ = 5.0 μM). All the synthesized derivatives have been subjected to docking studies related to soybean lipoxygenase. Compound **4g** exhibited a docking score of −9.2 kcal/mol and formed hydrophobic interactions with Val126, Tyr525, Lys526, Arg533, and Trp772, as well as a π−cation interaction with Lys526.

## 1. Introduction

**Scheme 1:**
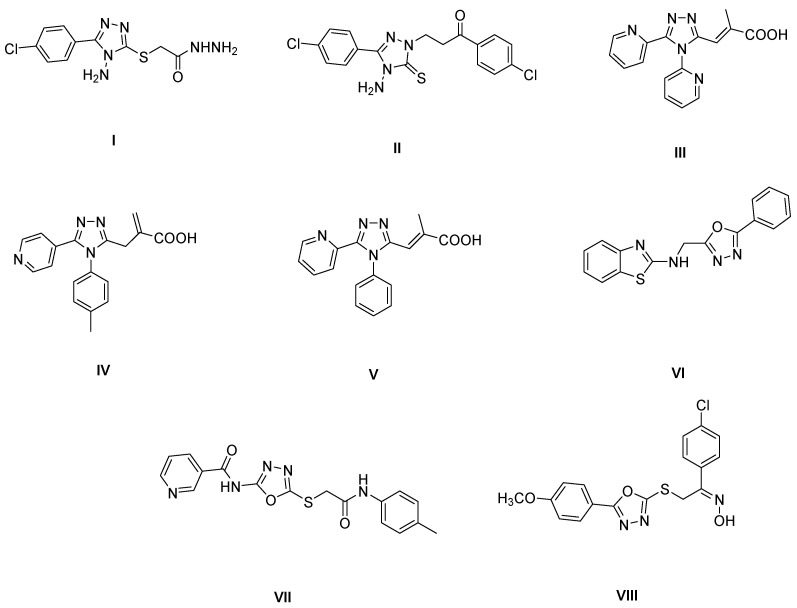
Structures of triazole and oxadiazole derivatives exhibiting potential anti-inflammatory activity (**I**–**VIII**) [2,3,4,6,7,9].

Triazoles are five-membered heterocyclic scaffolds bearing two carbon atoms and three nitrogen atoms [1]. Recent studies have found that compounds containing a triazole exhibit a wide range of biological activities, including antibacterial, antifungal, antiviral, antitumor, and anti-inflammatory properties (Scheme 1) [2,3,4]. Oxadiazole is a five-membered heterocyclic ring with two heteroatoms of nitrogen and one oxygen atom [5]. In addition, 1,3,4-oxadiazoles have been widely reported to exhibit a wide range of biological activities, including anti-inflammatory, anticancer, anti-HIV, antibacterial, and antimycobacterial properties (Scheme 1) [6,7,8,9].

**Scheme 2:**
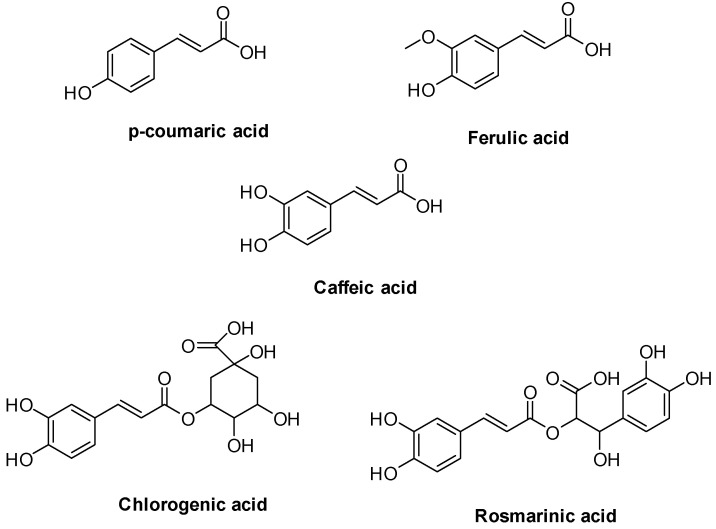
Chemical structures of bioactive cinnamic acid derivatives [14,15,16,17,18,19,20,21].

Cinnamic acid derivatives have been widely studied in recent years for their biological activities, such as antibacterial, antifungal, anti-inflammatory, neuroprotective, anticancer, and antidiabetic properties (Scheme 2). The type and location of the substituent groups in various cinnamic acid derivatives have been found to be linked to their biological activity [10]. They also suppress the activation of nitric oxide (NO), inducible nitric oxide synthase (iNOS), and cyclooxygenase-2 (COX-2) via the nuclear factor-kappa B (NF-κB) pathway. Additionally, a decrease in certain proinflammatory factors, such as tumor necrosis factor-α (TNF-α), interleukin-1 (IL-1), and nitric oxide (NO), was observed [11,12,13].

**Figure 1:**
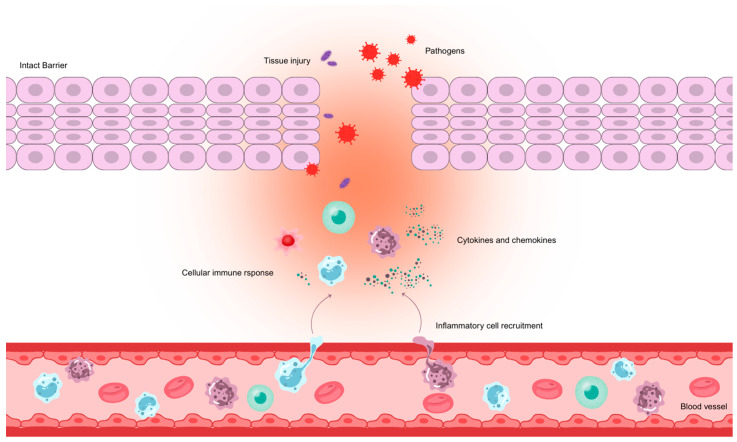
Overview of the inflammatory pathway activated by infection or tissue damage.

Inflammation is a dynamic process of the immune system and non-immune cells that is essential for survival during infection and tissue injury and is responsible for maintaining normal tissue homeostasis (Figure 1) [22,23,24]. Several enzymes like COX-2 and LOX, as well as factors such as cytokines, neutrophils, etc. are implicated in this phenomenon [25,26]. Furthermore, inflammation is implicated as a potential cause of different multifactorial diseases [25].

**Figure 2:**
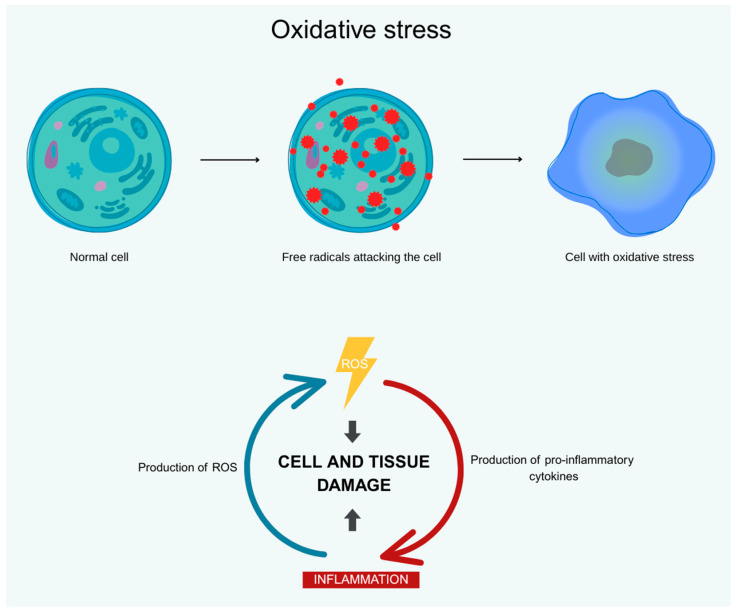
Free radicals induce oxidative damage to membrane lipids and proteins, leading to cellular component injury.

Oxidative stress is an imbalance between the increase in free radical species and the presence of antioxidant agents, which can lead to damage to cellular molecules such as DNA, proteins, and lipids (Figure 2) [27,28]. Oxidative stress is strongly associated with inflammation. Reactive oxygen species (ROS) derived from mitochondria activate signaling mediator molecules, such as the transcription factor nuclear factor kappa-B (NF-κB). NF-κB upregulates the production of inflammatory cytokines, including interleukin-1β (IL-1β) and tumor necrosis factor-α (TNF-α), as well as enzymes like inducible nitric oxide synthase (iNOS) and cyclooxygenase-2 (COX-2) [29].

Lipoxygenases are oxidative enzymes with non-heme iron atom in their active site and play a crucial role in inflammation [30,31,32]. Specifically, LOX catalyzes the process of arachidonic acid (AA) metabolism into leukotriene (LT), which facilitates the onset of inflammation [33]. 5-Lipoxygenase (5-LOX), implicated in various pathophysiological conditions such as atherosclerosis, psoriasis, and cancer, is particularly important due to its role in the production of leukotrienes [34].

**Scheme 3:**
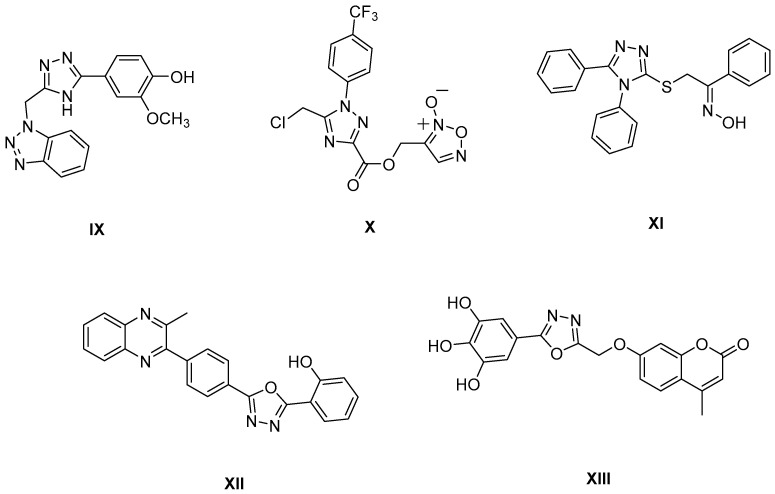
Representative five-membered heterocyclic scaffolds studied for their antioxidant and anti-inflammatory activities.

To deal with multifactorial diseases, it is necessary to develop molecules acting in different targets. One approach to achieve this is by synthesizing hybrid drugs, combining different moieties in a single multi-functional molecule, and offering multiple activities and better pharmacokinetic behaviors [35,36]. The hybrid design aims to enhance efficacy by enabling multi-target interactions, improving drug delivery to the site of action, increasing bioavailability, reducing toxicity, and overcoming resistance [37]. Notably, several hybrid molecules incorporating the 1,2,4-triazole moiety have been previously reported (Scheme 3) with significant antioxidant and anti-inflammatory activities, as demonstrated through the DPPH assay, COX-1/COX-2 inhibition assays, and the carrageenan-induced rat paw edema method [38,39,40]. Similarly, hybrids containing the 1,3,4-oxadiazole scaffold (Scheme 3) have also demonstrated comparable biological properties, as evaluated using the DPPH assay, carrageenan-induced rat paw edema, and cyclooxygenase inhibitor [41,42,43].

**Figure 3:**
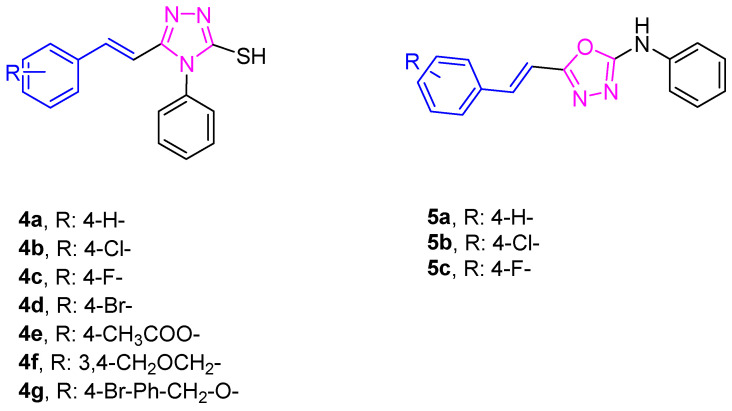
Hybrid molecules showing key pharmacophores, including cinnamic acid, 1,2,4-triazole, and 1,3,4-oxadiazole moieties.

In this study, cinnamic acid derivatives with 1,2,4-triazole and 1,3,4-oxadiazole (Figure 3) were synthesized and evaluated to enhance their antioxidant and anti-inflammatory properties by utilizing the beneficial effects of cinnamic acid derivatives, 1,2,4-triazole and 1,3,4-oxadiazole scaffolds. Fourteen novel compounds of substituted cinnamic acid derivatives linked with the 1,2,4-triazole pharmacophore, three linked with the 1,3,4-oxadiazole, and one with thiadiazole were designed, synthesized and evaluated in vitro for their ability to (i) interact with the stable DPPH free radical, (ii) inhibit the peroxidation of linoleic acid, and (iii) inhibit soybean lipoxygenase as a marker of anti-inflammatory activity. Finally, docking studies were conducted to predict binding interactions with lipoxygenase. Drug-likeness and lipophilicity were evaluated in silico.

## 2. Results

### 2.1. Chemistry

#### 2.1.1. Synthesis of Key Intermediates

**Scheme 4:**
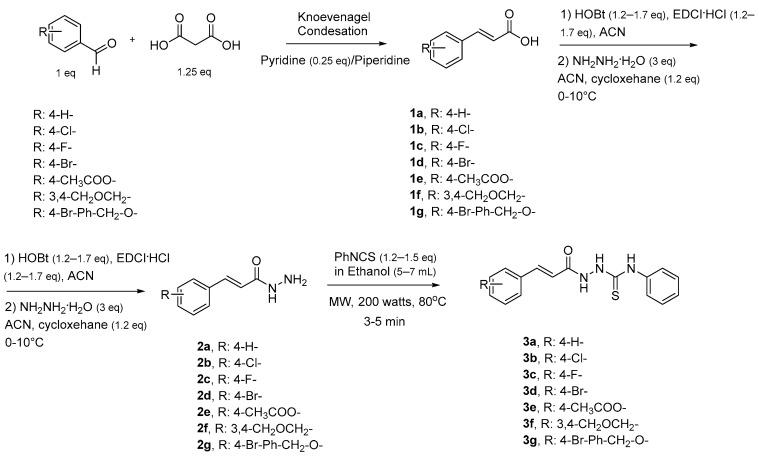
Synthesis of the key intermediates (**1a**–**g**), (**2a**–**g**), and (**3a**–**g**).

Starting materials (**1a**–**1g**) are obtained from commercial sources or synthesized via Knoevenagel condensation from the corresponding aldehyde and malonic acid in the presence of pyridine and piperidine (Scheme 4) [44,45].

For the esterification of the appropriate substituted cinnamic acid derivatives, 1-hydroxybenzotriazole (HOBt) and 1-ethyl-3-(3-dimethylaminopropyl)carbodiimide hydrochloride were added in anhydrous acetonitrile. The formation of the hydrazide derivatives (**2a**–**2g**) was achieved using hydrazine hydrate and the reaction was carried out in a mixed solvent system of acetonitrile and cyclohexane [46].

The cinnamoyl-thiosemicarbazide derivatives (**3a**–**3g**) were synthesized via the condensation between hydrazide derivatives and an appropriate amount of phenyl isothiocyanate in the presence of absolute ethanol as a solvent, under microwave irradiation [47,48,49].

#### 2.1.2. Synthesis of Target Molecules

**Scheme 5:**
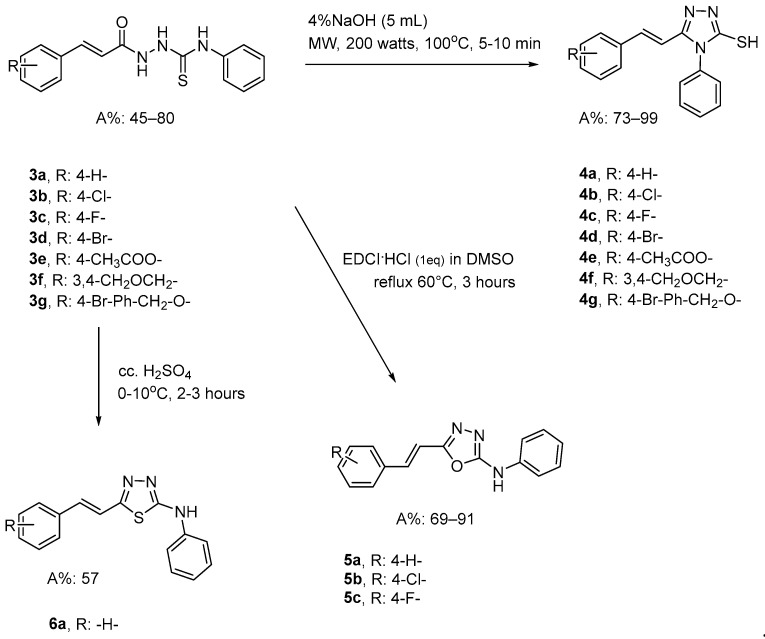
Synthesis of the target molecules **4a**–**g**, **5a**–**c**, and **6a**.

The synthesis of the final triazole derivatives (**4a**–**4g**) was conducted under basic conditions using a 4% sodium hydroxide (NaOH) solution, promoting intramolecular cyclization. The addition of hydrochloric acid yielded the final product (Scheme 5) [50]. For the synthesis of triazoles, the standard thermal methods were initially employed; however, the yields were relatively low [51]. To improve reaction yields, the conditions were optimized and subsequently employed microwave irradiation, which resulted in significantly better yields. The structures of the novel derivatives were confirmed using IR, ^1^H NMR, and ^13^C NMR. IR spectra presented characteristic absorption bands at 2600–2550 cm^−1^ (S-H bond stretch) and 1690–1630 cm^−1^ (N=C bond stretch). For the ^1^H NMR data, the proton of SH was recorded at δ 13.7 ppm. The *trans* stereochemistry of the double bond was confirmed by the coupling constant (*J* = 16.4 Hz) observed in the ^1^H NMR spectrum. For the ^13^C NMR, the carbons of the heterocycle ring appear at δ 167.9 ppm and 149 ppm. The absence of tautomeric forms involving the SH groups in the synthesized compounds was supported by the NMR data, which showed no evidence of tautomerism. This observation is further consistent with previous findings reported in the literature [51].

For the synthesis of the final oxadiazole derivatives (**5a**–**5c**), 1-ethyl-3-(3-dimethylaminopropyl)carbodiimide hydrochloride was added to cinnamoyl-thiosemicarbazide derivatives in dimethyl sulfoxide (DMSO) and heated under reflux. The structures of the novel derivatives were confirmed using IR, ^1^H NMR and ^13^C NMR, and spectroscopy. IR spectra presented the characteristic absorption bands at 3000–2800 cm^−1^ (N-H bond stretch) and 1600–1500 cm^−1^ (C=C bond stretch). As for the ^1^H NMR data, the proton of NH was recorded at δ 10.6 ppm. The double bond was determined to have a trans configuration based on the coupling constant (*J* = 16.3 Hz) observed in the ^1^H NMR spectrum. As for the ^13^C NMR, the C-2 and C5-carbons of the oxadiazole ring appear in the range of 159–160 ppm and 157–159 ppm, respectively.

For the synthesis of the 1,3,4-thiadiazole derivative (**6a**), an appropriate cinnamoyl-thiosemicarbazide derivative was added gradually to concentrated sulfuric acid under stirring at 0 °C for 2 h. After the completion of the reaction, the mixture is poured into ice, and the resulting solid is filtered and washed several times with water. Finally, it is recrystallized from methanol/water. IR spectra presented the characteristic absorption bands at 3000–2800 cm^−1^ (N-H bond stretch) and 1600–1500 cm^−1^ (C=C bond stretch). As for the ^1^H NMR data, the proton of NH was recorded at δ 10.3 ppm and the trans stereochemistry of the double bond was confirmed by the coupling constant (*J* = 16.4 Hz) observed in the ^1^H NMR spectrum. For the ^13^C NMR, the C-2 and C-5 carbons of the thiadiazole ring appear at 183.6 ppm and 168.1 ppm, respectively. The synthesis of oxadiazoles and thiadiazoles under standard conditions already provided excellent yields. Therefore, further optimization or the application of microwave-assisted methods was not deemed necessary.

All compounds were obtained in good to excellent yields. For compounds **3a**–**3g** yields vary from 45% to 91%, for compounds **4a**–**4g** between 73% and 99%, for compounds **5a**–**5c** between 69% and 91%, and compound **6a** was obtained with a 57% yield. Compounds **4a**–**4g** afforded excellent yields under microwave irradiation except for compound **4g** (73%) bearing the bulkier substituent. While in series **5a**–**5c** all the substituted derivatives presented good yields, while the non-substituted one presented the lower one. The final products **3a**–**3g** and **4a**–**4g** were purified by recrystallization from ethanol and compound **5a** was recrystallized from methanol/water. Compounds **3a**, **4d**, **5a**, and **6a** have been previously reported in the literature [52,53,54,55]. For all the compounds, the identification was carried out by melting point, IR, ^1^H NMR, and ^13^C NMR.

### 2.2. Physicochemical Studies

#### 2.2.1. Determination of Lipophilicity

Lipophilicity is one of the principal properties that describes the activity and pharmacokinetic behavior of a drug candidate related to solubility and the membrane’s permeability influencing the ADMET profile (absorption, distribution, metabolism, elimination, and toxicity) of a bioactive molecule [31,56]. The Bio-Loom program of BioByte Corp (Claremont, CA, USA, http://www.biobyte.com/) was used to theoretically calculate the lipophilicity as clog *P* values. According to the calculated clog *P* values, the most lipophilic compound is **4g** (clog *P* = 7.08), while the less lipophilic one is **3e** (clog *P* = 1.74).

#### 2.2.2. Theoretical Calculation of Physicochemical Properties

Drug-likeness is a qualitative assessment of a molecule’s potential to become an oral drug in terms of bioavailability. This assessment can be reached from the molecular structure, before the molecule’s synthesis and its biological evaluation. Many in silico platforms have been developed to predict ADMET properties. One of them is the online platform Molinspiration (www.molinspiration.com, Molinspiration Cheminformatics) (accessed on 4 May 2025). This platform uses Lipinski’s Rule of Five for the evaluation of drug-likeness. The calculated values include miLog P, which indicates lipophilicity; TPSA, the topological polar surface area, which is a very good descriptor for drug absorption, including intestinal absorption, bioavailability, Caco-2 permeability, and blood-brain barrier penetration; Natoms, the number of atoms in the molecule; MW, the molecular weight, which influences absorption, distribution, and overall drug-likeness; nON, the number of hydrogen bond acceptors; nOHNH, the number of hydrogen bond donors, important for hydrogen bonding capacity and interaction with biological targets; Nviolations, the number of Lipinski’s rule of five violations; Nrotb, the number of rotatable bonds, a topological parameter measuring molecular flexibility and a good predictor of oral bioavailability of drugs; and Volume, the molecular volume, which reflects the spatial size of the molecule and influences membrane permeability and steric interactions with biological targets. All the synthesized compounds do not present any violation, except for compounds **3g** (clog *P* = 4.94) and **4g** (clog *P* = 7.08). The results are presented in Table 1.

### 2.3. Biological Evaluation

In this study, the synthesized derivatives were tested in vitro for their capacity to interact with the stable free radical DPPH, inhibit AAPH-induced peroxidation of linoleic acid, and soybean lipoxygenase.

Oxidative stress is a molecular-level cellular process occurring when excessive reactive oxygen species (ROS) surpass the ability of antioxidant defense mechanisms. Endogenous and exogenous antioxidants, enzymatic or non-enzymatic, are necessary to prevent or show cellular damage by scavenging free radicals [57].

Firstly, the synthesized derivatives were evaluated for their ability to interact with the stable free radical 2,2-diphenyl-1-picrylhydrazyl (DPPH) at a concentration of 100 μM after 20 and 60 min (Table 2). DPPH is a valuable tool for studying antioxidant activity. Its reactivity with lipophilic compounds is not solely determined by their lipophilicity. The specific chemical structure of the antioxidant, the solvent used, and other factors can all influence the interaction with DPPH. However, lipophilic compounds tend to be well dissolved in organic solvents reacting with DPPH. Based on previous findings [58], the antioxidant activity is related with lipophilicity. In this assay, nordihydroguaiaretic acid (NDGA) was used as a reference standard [59].

Among the cinnamoyl-thiosemicarbazide derivatives (**3a**–**3g**), compounds **3a**, **3b**, **3c**, **3d**, **3f**, and **3g** exhibited excellent DPPH radical scavenging activity at 60 min, with compound **3d** (Br-substituted derivative) showing the highest activity (97%) comparable to NDGA (93%). The interaction of compounds **3a**–**3g** with the DPPH radical seems to increase over time, except for compounds **3a**, **3d**, and **3f**. Compound **3c**, bearing a 4-F substitution, presents high interaction at 20 min, 90% which seems to be maintained over time at 91%. The 4F-substitution benefits from low molar refractivity (MR_F_ = 0.092), which may facilitate better access to the DPPH free radical and enhance its interaction. It is noteworthy that compound **3e** with the lowest lipophilicity (clog *P* = 1.74) shows a dramatic decrease in DPPH radical interaction, from 83% at 20 min to just 21% at 60 min.

Among the triazole derivatives, compounds **4a**, **4c**, **4e**, and **4g** demonstrated potent interaction with DPPH apart from compound **4d** which presents medium interaction at 20 and 60 min, and **4b** which presents low interaction at 20 min. For compounds **4a**–**4g**, interaction with the DPPH radical generally decreases over time, apart from compounds **4a**, **4b**, and **4c**. Compounds **4e** and **4g** exhibit the highest DPPH scavenging activity over time (20 min and 60 min) comparable to NDGA which was used as a reference compound. However, this activity does not appear to be directly attributed to the cinnamoyl-triazole moiety. In general, cyclization to form the hybrid cinnamoyl-triazole structure favors the interaction with the DPPH radical at 60 min, except for compounds **3d**, **4d**, and **3f**, **4f**.

Additionally, oxadiazole derivatives **5b** and **5c** showed good interaction with the DPPH radical. Compound **5b** exhibited an increase in activity over time, whereas compound **5c** showed a decrease in DPPH interaction at 60 min. Compound **5c** presents analogous activity to the standard drug, NDGA. It appears that the triazole moiety contributes more significantly to the interaction with the DPPH radical. This is obvious considering the greater increase in activity observed from the % interaction values for compounds **4a**, **4b**, and **4c** in comparison to the corresponding compounds **5a**, **5b**, and **5c**. The interaction increases over time for compounds **3a**, **4a**, and **6a**. The increase in lipophilicity, clog *P* (**3a**) < clog *P* (**4a**) < clog *P* (**6a**), is associated with enhanced activity. The presence of a thiadiazole ring in compound **6a** is related significantly with higher antioxidant activity compared to the oxadiazole ring of compound **5a**.

In the lipid peroxidation assay, 2,2′-azobis(2-methylpropionamidine)dihydrochloride (AAPH) was used as a peroxyl radical inducer, and sodium linoleate as a substrate. The formation of 13-hydroperoxy-linoleic acid from linoleic acid was monitored by measuring absorbance at 234 nm. Trolox was used as a reference compound.

Most of the cinnamoyl-thiosemicarbazide and triazole derivatives exhibited moderate to low inhibitory activity, except for compounds **3g**, **4c**, and **4e**. Among the oxadiazole derivatives, only compound **5c** demonstrated moderate activity.

Among the cinnamoyl-thiosemicarbazide derivatives **3a**–**3g**, compound **3g**, which exhibits the highest inhibitory activity, also possesses the highest lipophilicity (clog *P* = 4.94) analogous to Trolox, the reference compound. In contrast, compound **3c**, which contains an F-substituent, shows the lowest activity likely associated with its low molar refractivity of the 4-substituent (MR_F_ = 0.092).

Among the triazole derivatives, compound **4c** (R = 4–F) exhibited the highest inhibitory effect comparable to Trolox, followed by compound **4e** (R = 4–CH_3_COO–). All other triazole derivatives showed moderate inhibitory activity apart from compound **4d** bearing a 4-Br, which presents 20% inhibition. Based on the results, there does not appear to be a clear correlation between inhibitory activity and lipophilicity. Cyclization of compounds **3a**–**3g** into cinnamoyl-triazole derivatives resulted in an increased lipid peroxidation inhibitory activity for compounds **4c**, **4e**, and **4f**. In contrast, a decrease in inhibitory activity was observed for compounds **4a**, **4b**, **4d**, and **4g**. This cannot be explained by any special physicochemical characteristic.

The triazole moiety appears to contribute to the lipid peroxidation inhibition, due to the higher activity observed in compounds **4a**, **4b**, and **4c** compared to the oxadiazole derivatives **5a**, **5b**, and **5c**. Perhaps this can be correlated with the increase in lipophilicity. Cyclization of compound **3a** to the thiadiazole derivative, compound **6a**, results in a slight reduction in inhibitory activity, probably due to increased lipophilicity (clog *P*_3a_ = 2.39 vs. clog *P*_6a_ = 4.76). The thiadiazole ring, compared to the triazole ring, appears to contribute more significantly to the inhibitory activity, as observed from the comparison of compounds **4a** and **6a**. It seems that again lipophilicity plays an important role (clog *P*_4a_ = 4.53 vs. clog *P*_6a_ = 4.76). The thiadiazole ring, compound **6a**, contributes significantly to lipid peroxidation inhibition compared to the oxadiazole ring, compound **5a** (clog *P*_5a_ = 3.93 vs. clog *P*_6a_ = 4.76).

Lipoxygenase (LOX) is responsible for the formation of leukotrienes, important inflammatory mediators. Due to its important role, it is considered as a therapeutic target for inflammatory diseases. Soybean lipoxygenase (LOX), a plant-derived enzyme, has been extensively utilized as a model for investigating the functional and structural properties of the lipoxygenase family [34,60], due to its significant homology with human 5-LOX [35,61]. The novel derivatives have been evaluated for soybean lipoxygenase inhibition (Table 2). Based on the IC_50_ values, the triazole derivatives appear to exhibit a stronger inhibitory effect compared to the cinnamoyl-thiosemicarbazide derivatives. All of the synthesized compounds exhibited excellent sLOX inhibition, except for compounds **3b**, **3c**, and **4c**, which presented medium inhibition 35–49% at 100 µM. Among the cinnamoyl-thiosemicarbazide derivatives, compound **3d** (R = 4–Br) presented the best activity. For triazole derivatives, compounds **4b** and **4g** possess the highest inhibitory effect (4.5 µM) against sLOX followed by compound **4e** (5.5 µM). This cannot be explained by a special physicochemical characteristic. Cyclization of compounds **3a**–**3g** into cinnamoyl-triazole derivatives generally led to an increase in sLOX inhibition, with the only exceptions being compounds **4c** and **4d**, which showed lower activity compared to **3c** and **3d**. Among the series **5a**–**5c**, compound **5c**, the F-substituted derivative, presented the highest activity. NDGA was used as a reference drug.

Lipophilicity and molar refractivity (MR) do not appear to play a significant role in the inhibitory activity against sLOX. Lipophilicity is considered one of the most important parameters with regard to LOX inhibitors according to the literature [62]. However, no role for this property was found to exist for these compounds. No correlation can be found between the antioxidant and lipoxygenase inhibition.

### 2.4. Docking Simulation Soybean Lipoxygenase Studies

Docking studies were performed on soybean lipoxygenase-1 (PDB ID: 3PZW). The crystal structure of soybean lipoxygenase-1 (PDB ID: 3PZW) does not include a co-crystallized ligand, requiring the identification of possible allosteric binding sites beyond the known iron-binding and substrate-binding regions, as noted in recent studies. Detsi, A. et al. [63] have identified using SiteMap’s three potential binding sites [64]. Researchers have found that Site 1 is located between the amino-terminal β-barrel (PLAT domain) and the α-helical domain, which houses the catalytic iron. Sites 2 and 3 are also positioned within the α-helical domain. Additionally, Site 1 is recognized as a potential binding site in blind docking studies [63,65,66].

**Figure 4:**
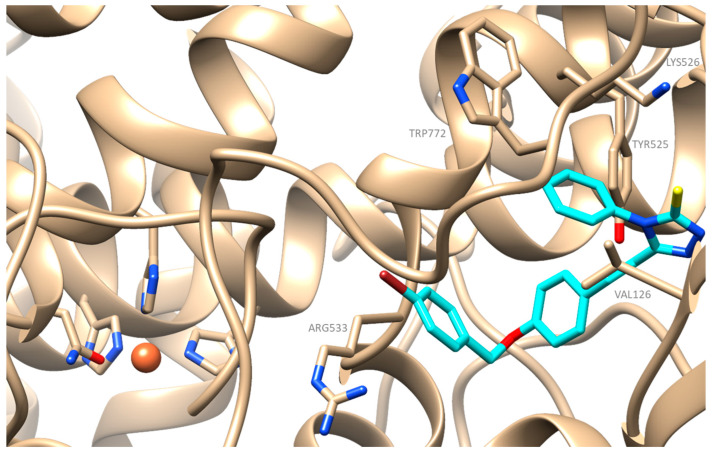
The 3D preferred docking pose of compound **4g** (depicted in cyan) bound to soybean lipoxygenase. Iron appears as an orange sphere.

**Figure 5:**
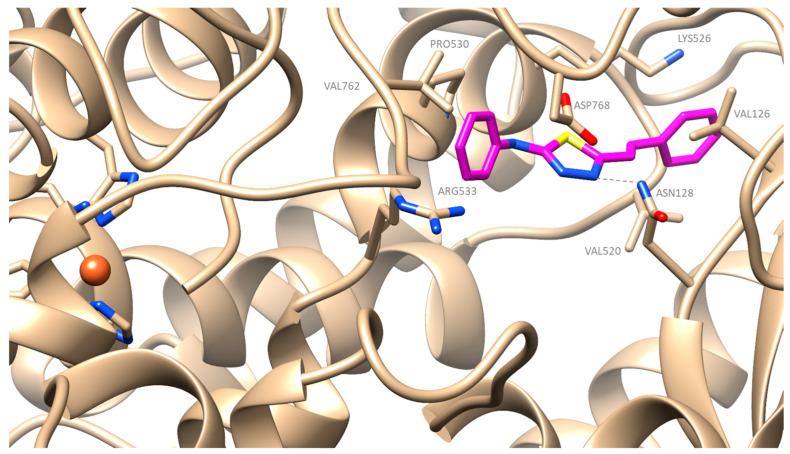
The 3D preferred docking pose of compound **6a** (depicted in magenta) bound to soybean lipoxygenase. The one hydrogen bond is illustrated with dashed gray lines. Iron appears as an orange sphere.

**Figure 6:**
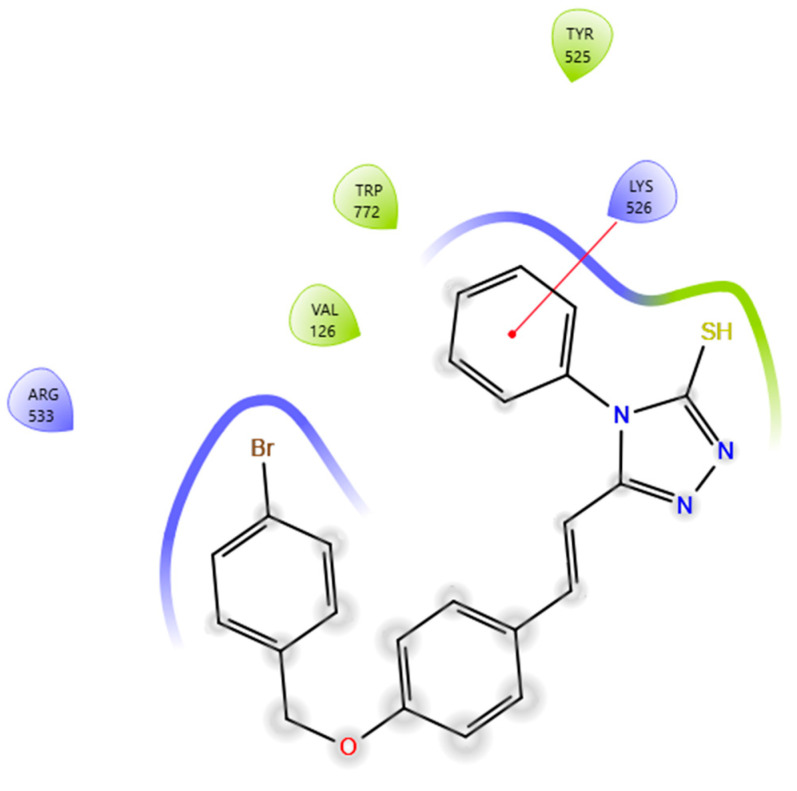
Ligand interaction diagram of compound **4g** to soybean lipoxygenase (ID: 3PZW). The π−cation interaction is presented with a red line. The hydrophobic residues are shown in green, the polar ones in cyan, and the positive charged in blue. The figure is made with free Maestro [67]. (Free Maestro academic license—Schrödinger Release 2025-2: Maestro, Schrödinger, LLC, New York, NY, USA, 2025).

**Figure 7:**
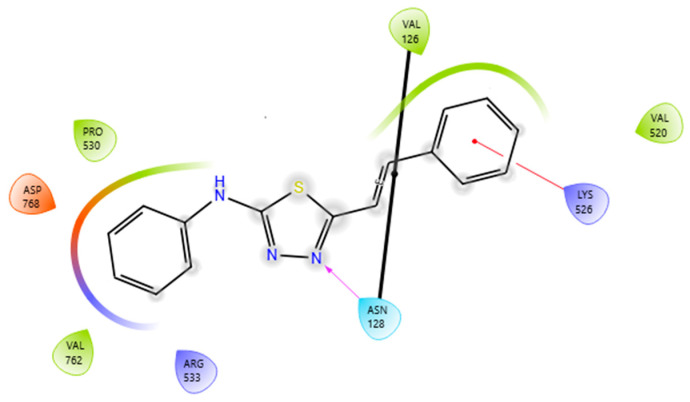
Ligand interaction diagram of compound **6a** to soybean lipoxygenase (ID: 3PZW). The hydrogen bond interaction with residues is illustrated by a purple dashed arrow and the π−cation interaction is presented with a red line. The hydrophobic residues are shown in green, the polar ones in cyan, the positive charged in blue, and the negative charged in red. The figure is made with free Maestro [67]. (Free Maestro academic license—Schrödinger Release 2025-2: Maestro, Schrödinger, LLC, New York, NY, USA, 2025).

Based on the above research studies, blind docking was performed in addition to that of the active center in order to include all potential binding sites. It has been concluded that compounds bind to Site 1 being in accordance with the above research studies. Compounds **4b**, **4g**, and **6a** were the most potent among the series. Compound **4b** presents a binding score of −7.8 kcal/mol, while compound **4g** a score of −9.2 kcal/mol and compound **6a** a score of −8.6 kcal/mol. Compound **4b** develops hydrophobic interactions with the amino acids Val126, Val520, Lys526, Trp772, while compound **4g** develops hydrophobic interactions with amino acids. Val126, Tyr525, Lys526, Arg533, Trp772, and a π−cation interaction with Lys526 (Figure 4). Compounds **4b** and **4g** seem to present a similar binding mode to the enzyme. Compound **6a** seems to interact hydrophobically with amino acids Val126, Val520, Pro530, Arg,533, Val762, Asp768. Moreover, it interacts with π−cation interaction with Lys526 and with a hydrogen bond with Asn128 (Figure 5). Ligand interaction diagrams are illustrated in Figure 6 and Figure 7.

## 3. Experimental Section

### 3.1. Materials and Instruments

All chemicals, solvents, chemical and biochemical reagents were of analytical grade and purchased from commercial sources (Merck, Merck KGaA, Darmstadt, Germany, Fluka Sigma-Aldrich Laborchemikalien GmbH, Hannover, Germany, Alfa Aesar, Karlsruhe, Germany and Sigma St. Louis, MO, USA). Soybean lipoxygenase, sodium linoleate, arachidonic acid (AA), and 2,2-azinobis-2-methyl-propanimidamine HCl (AAPH) were obtained from Sigma Aldrich, Merck KGaA, Darmstadt, Germany. Nordihydroguaiaretic acid (NDGA), 1,1-diphenyl-2-picrylhydrazyl (DPPH), and 6-hydroxy-2,5,7,8-tetramethyl-chroman2-carboxylic acid (Trolox) were purchased from the Sigma-Aldrich Chemical Co. (St. Louis, MO, USA). All starting materials were used without further purification.

Melting points (uncorrected) were determined on a MEL-Temp II (Lab. Devices, Holliston, MA, USA). For the in vitro tests, UV-Vis spectra were obtained on a Perkin-Elmer UV/Vis Spectrometer Lamda 20 (Perkin-Elmer Corporation Ltd., Lane Beaconsfield, Bucks, UK). Infrared spectra (KBr pellets) were recorded with a Perkin-Elmer spectrum BX FT-IR spectrometer (Perkin-Elmer Corporation Ltd., Lane Beaconsfield, Bucks, UK). The CEM Discover Microwave apparatus (CEM Corporation, a company headquartered in Matthews, NC, USA) was used for the reactions.

The ^1^H Nucleic Magnetic Resonance (^1^H NMR) spectra were recorded at 500 MHz in DMSO-d_6_ on an Agilent 500/54 (DD2, Santa Clara, CA, USA) using tetramethylsilane as an internal standard unless otherwise stated. Agilent 500/54 (DD2) ^13^C NMR spectra were obtained at 125 MHz in DMSO-d_6_ solutions with tetramethylsilane as the internal reference unless otherwise stated. Chemical shifts are expressed in ppm and coupling constants *J* in Hz. Reactions were monitored by thin-layer chromatography on 5554 F_254_ Silica gel/TLC cards (Merck and Fluka Chemie GmbH Buchs, Steinheim, Switzerland). 

### 3.2. Chemistry General Procedures and Characterization Data

#### 3.2.1. Synthesis of α,β- Unsaturated Carboxylic Acids (**1a**–**1g**)

Malonic acid (1.25 equiv) was dissolved in pyridine (0.25 equiv), and then the appropriate substituted aldehyde (1 equiv) and a catalytic amount of piperidine were added to the mixture and heated under reflux for approximately 1 h with monitoring by TLC. After completion of the reaction, the mixture was poured into ice, and 2 M HCl acid was added and cooled. Once a precipitate was formed, it was collected by filtration and from recrystallized ethanol/water to give the corresponding pure α,β-unsaturated carboxylic acid.

#### 3.2.2. General Method for the Synthesis of Cinnamoyl-Thiosemicarbazide Derivatives (**3a**–**3g**)

Substituted cinnamic acids were dissolved in acetonitrile and then HOBt or N-Hydroxysuccinimide (NHS) (1.2–1.7 equiv) and EDCI· HCl (1.2–1.7 equiv) were added to the mixture. The reaction mixture was stirred for 2 h. After that, the mixture was cooled to 0–10 °C and a solution of NH_2_NH_2_·H_2_O (3 equiv), cyclohexane (1.2 equiv), and acetonitrile was added. After 30 min, water was poured into the mixture, and the resulting solution was extracted with ethyl acetate. The organic layers were collected, combined and subsequently washed with 10% sodium carbonate and brine, dried over anhydrous Na_2_SO_4_, and concentrated.

The resulting mixture was dissolved in 5–7 mL of absolute ethanol in an appropriate amount of phenyl isothiocyanate, then (1.2–1.5 eq) was added and the reaction proceeded using microwave irradiation at a power of 200 watts, and temperature of 80 °C for 3–5 min. The formed precipitate is cooled, filtered under vacuum, washed with ether, and recrystallized from absolute ethanol.

*2-Cinnamoyl-N-phenylhydrazine-1-carbothioamide* (**3a**): The spectral data were in agreement with the literature data [55].
*(E)-2-(3-(4-Chlorophenyl)acryloyl)-N-phenylhydrazine-1-carbothioamide* (**3b**): Yield: 74%, yellow solid, m.p.: 208–209 °C (recrystallization from C_2_H_5_OH). IR (KBr, cm^−1^): 3282 (N-H), 3227 (N-H), 3088 (N-H), 1651 (C=O), 1624 (C=C), 1362. ^1^H NMR (500 MHz, DMSO-d_6_): δ 10.24 (s, 1H, -NH), 9.76 (brs, 2H, -NH-NH-), 7.64 (d, *J* = 8.5 Hz, 2H, ArH), 7.56 (d, *J* = 15.8 Hz, 1H, -CH=CH-), 7.51 (d, *J* = 8.4 Hz, 2H, -ArH), 7.48–7.40 (m, 2H, ArH), 7.33 (t, *J* = 7.7 Hz, 2H, ArH), 7.15 (t, *J* = 7.4 Hz, 1H, ArH), 6.66 (d, *J* = 15.8 Hz, 1H, -CH=CH-). ^13^C NMR: (126 MHz, DMSO): δ 180.8, 139.2, 138.6, 134.3, 133.6, 129.4, 129.2, 128.1, 126.2, 125.4, 120.7.
*(E)*-2-(3-(4-Fluorophenyl)acryloyl)-N-phenylhydrazine-1-carbothioamide (**3c**): Yield: 45%, yellow solid, m.p.: 202–203 °C (recrystallization from C_2_H_5_OH). IR (KBr, cm^−1^): 3287 (N-H), 3223 (N-H), 2981 (N-H), 1652 (C=O), 1626 (C=C), 1363. ^1^H NMR (500 MHz, DMSO-d_6_): δ 10.22 (s, 1H, NH), 9.78 (s, 1H, -NH-NH-), 9.72 (s, 1H, -NHNH-), 7.68 (dd, *J* = 8.5 Hz, 2H, ArH), 7.55 (d, *J* = 15.9 Hz, 1H, -CH=CH-), 7.50–7.40 (m, 2H, ArH), 7.29 (dt, *J* = 8.3 Hz, 4H, ArH), 7.15 (t, *J* = 7.3 Hz, 1H, ArH), 6.61 (d, *J* = 15.9 Hz, 1H, -CH=CH-). ^13^C NMR: (126 MHz, DMSO): δ 181.0, 164.0, 162.1, 139.3, 139.0, 131.4, 130.1, 128.3, 126.1, 125.2, 119.9, 116.2.
*(E)*-2-(3-(4-Bromophenyl)acryloyl)-N-phenylhydrazine-1-carbothioamide (**3d**): Yield: 79%, yellow solid, m.p.: 209–210 °C (recrystallization from C_2_H_5_OH). IR (KBr, cm^−1^): 3282 (N-H), 3232 (N-H), 2981 (N-H), 1652 (C=O), 1623 (C=C), 1362. ^1^H NMR (500 MHz, DMSO-d_6_): δ 10.24 (s, 1H, NH), 9.77 (brs, 2H, -NH-NH-), 7.70–7.56 (m, 4H, ArH), 7.54 (d, *J* = 16.1 Hz, 1H, -CH=CH-), 7.45 (d, *J* = 7.7, 2H, ArH), 7.32 (t, *J* = 7.7 Hz, 2H, ArH), 7.15 (t, *J* = 7.3 Hz, 1H, ArH), 6.67 (d, *J* = 16.2 Hz, 1H, -CH=CH-). ^13^C NMR: (126 MHz, DMSO): δ 180.8, 180.4, 139.2, 138.7, 133.9, 132.1, 129.6, 128.1, 125.9, 1250, 123.1, 120.8.
*(E)*-4-(3-Oxo-3-(2-(phenylcarbamothioyl)hydrazineyl)prop-1-en-1-yl)phenyl acetate (**3e**): Yield: 46%, yellow solid, m.p.: 183–184 °C (recrystallization from C_2_H_5_OH). IR (KBr, cm^−1^): 3280 (N-H), 3219 (N-H), 2981 (N-H), 1650 (C=O), 1623 (C=C), 1366. ^1^H NMR (500 MHz, DMSO-d_6_): δ 10.22 (s, 1H, NH), 9.77 (s, 1H, -NH-NH-), 9.72 (s, 1H, -NHNH-), 7.66 (d, *J* = 8.3 Hz, 2H, ArH), 7.57 (d, *J* = 15.9 Hz, 1H, -CH=CH-), 7.46 (d, *J* = 7.3 Hz, 2H, ArH), 7.33 (t, *J* = 7.8 Hz, 2H, ArH), 7.21 (d, *J* = 8.3 Hz, 2H, ArH), 7.15 (t, *J* = 7.5 Hz, 1H, ArH), 6.63 (d, *J* = 15.9 Hz, 1H, -CH=CH-), 2.28 (s, 3H, CH_3_). ^13^C NMR: (126 MHz, DMSO): δ 180.8, 169.1, 151.5, 139.5, 139.2, 139.0, 132.3, 128.9, 128.1, 125.8, 125.0, 122.6, 120.0, 20.9.
*(E)*-2-(3-(Benzo[d][1,3]dioxol-5-yl)acryloyl)-N-phenylhydrazine-1-carbothioamide (**3f**): Yield: 45%, yellow solid, m.p.: 183–184 °C (recrystallization from C_2_H_5_OH). IR (KBr, cm^−1^): 3283 (N-H), 3227 (N-H), 2981 (N-H), 1650 (C=O), 1625 (C=C), 1356. ^1^H NMR (500 MHz, DMSO-d_6_): δ 10.10 (s, 1H, NH), 9.74 (s, 1H, -NH-NH-), 9.71 (s, 1H, -NHNH-), 7.47 (t, *J* = 15.4, 8.7 Hz, 3H, -CH=CH-, ArH), 7.32 (t, *J* = 7.7 Hz, 2H, ArH), 7.19 (s, 1H, ArH), 7.14 (dd, *J* = 7.8 Hz, 2H, ArH), 6.98 (d, *J* = 7.9 Hz, 1H, ArH), 6.49 (d, *J* = 15.8 Hz, 1H, -CH=CH-), 6.08 (s, 2H, -CH_2_). ^13^C NMR: (126 MHz, DMSO): δ 180.8, 148.8, 148.0, 139.8, 139.2, 129.0, 128.0, 125.9, 125.0, 123.6, 117.9, 108.7, 106.3, 101.6.
*(E)*-2-(3-(4-((4-Bromobenzyl)oxy)phenyl)acryloyl)-N-phenylhydrazine-1-carbothioamide (**3g**): Yield: 78%, yellow solid, m.p.: 207–208 °C (recrystallization from C_2_H_5_OH). IR (KBr, cm^−1^): 3248 (N-H), 2981 (N-H), 2160, 1591 (C=O), 1361. ^1^H NMR (500 MHz, DMSO-d_6_): δ 10.11 (s, 1H, NH), 9.74 (s, 1H, -NH-NH-), 9.71 (s, 1H, -NHNH-), 7.60 (d, *J* = 8.1 Hz, 2H, ArH), 7.57 (d, *J* = 8.4 Hz, 2H, ArH), 7.51 (d, *J* = 15.9 Hz, 1H, -CH=CH-), 7.44 (dd, *J* = 8.1, 7.5 Hz, 4H, ArH), 7.32 (t, *J* = 7.7 Hz, 2H, ArH), 7.15 (t, *J* = 7.4 Hz, 1H, ArH), 7.07 (d, *J* = 8.4 Hz, 2H, ArH), 6.51 (d, *J* = 15.8 Hz, 1H, -CH=CH-), 5.14 (s, 2H, CH_2_). ^13^C NMR: (126 MHz, DMSO): δ 180.8, 159.5, 139.6, 139.2, 136.3, 131.4, 129.9, 129.4, 128.0, 127.5, 125.9, 124.9, 121.1, 117.6, 115.4, 68.5.

#### 3.2.3. General Method for the Synthesis of Triazole-Cinnamic Acid Derivatives (**4a**–**4g**)

For the synthesis of the final triazole derivatives, the intermediate cinnamoyl-thiosemicarbazide derivative and 5 mL of 4% sodium hydroxide (NaOH) solution were irradiated at 200 Watts and 100 °C for approximately 5–10 min. After completion of the reaction, 2Μ HCl was added to the reaction mixture until a precipitate was formed. The precipitate was filtered, washed with water, and left under vacuum to dry. Finally, the precipitate is recrystallized from absolute ethanol.

*(E)*-4-Phenyl-5-styryl-4H-1,2,4-triazole-3-thiol (**4a**): Yield: 89%, white crystal solid, m.p.: 259–260 °C (recrystallization from C_2_H_5_OH). IR (KBr, cm^−1^): 2690 (S-H), 1634 (N=C), 1544, 1339, 1295 (C-N). ^1^H NMR (500 MHz, DMSO-d_6_): δ 14.04 (s, 1H, SH), 7.65–7.54 (m, 3H, ArH), 7.48–7.41 (m, 4H, ArH), 7.35 (d, *J* = 8.0 Hz, 2H, ArH), 7.31 (d, *J* = 16.4 Hz, 2H, -CH=CH-, ArH), 6.46 (d, *J* = 16.4 Hz, 1H, -CH=CH-). ^13^C NMR (126 MHz, DMSO-d_6_): δ 167.8, 149.1, 136.2, 134.8, 133.6, 129.6, 129.6, 129.5, 128.9, 128.5, 127.2, 111.0.
*(E)*-5-(4-Chlorostyryl)-4-phenyl-4H-1,2,4-triazole-3-thiol (**4b**): Yield: 99%, white solid, m.p.: 267–268 °C (recrystallization from C_2_H_5_OH). IR (KBr, cm^−1^): 2638 (S-H), 1591(N=C), 1547, 1290 (C-N). ^1^H NMR (500 MHz, DMSO-d_6_): δ 13.22 (s, 1H, SH), 6.76 (dq, *J* = 7.1 Hz, 3H, ArH), 6.63 (dd, *J* = 7.5 Hz, 4H, ArH), 6.56 (d, *J* = 8.2 Hz, 2H, ArH), 6.49 (d, *J* = 16.4 Hz, 1H, -CH=CH-), 5.64 (d, *J* = 16.4 Hz, 1H, -CH=CH-).^13^C NMR (126 MHz, DMSO-d_6_): δ 167.9, 149.0, 134.8, 133.9, 133.7, 133.5, 129.6, 129.6, 129.0, 128.9, 128.5, 111.8.
*(E)*-5-(4-Fluorostyryl)-4-phenyl-4H-1,2,4-triazole-3-thiol (**4c**): Yield: 99%, white solid, m.p.: 240–241 °C (recrystallization from C_2_H_5_OH). IR (KBr, cm^−1^): 2615 (S-H), 1636 (N=C), 1546, 1297 (C-N). ^1^H NMR (500 MHz, DMSO-d_6_): δ 13.19 (s, 1H, SH), 6.78–6.74 (m, 1H, ArH), 6.74–6.71 (m, 2H, ArH), 6.67 (dd, *J* = 7.7 Hz, 2H, ArH), 6.60 (dd, *J* = 6.6 Hz, 2H, ArH), 6.48 (d, *J* = 16.4 Hz, 1H, -CH=CH-), 6.32 (t, J = 8.9 Hz, 2H, ArH), 5.56 (d, *J* = 16.4 Hz, 1H, -CH=CH-).^13^C NMR (126 MHz, DMSO-d_6_): δ 167.8, 163.7, 161.7, 149.2, 135.0, 133.5, 131.5, 129.6, 128.5, 116.0, 115.8, 110.9.
*(E)*-5-(4-Bromostyryl)-4-phenyl-4H-1,2,4-triazole-3-thiol (**4d**): The spectral data were in agreement with the literature data [54].
*(E)*-4-(2-(5-Mercapto-4-phenyl-4H-1,2,4-triazol-3-yl)vinyl)phenyl acetate (**4e**): Yield: 91%, white solid, m.p.: 265–266 °C (recrystallization from C_2_H_5_OH). IR (KBr, cm^−1^): 2658 (S-H), 1635 (N=C), 1543, 1299 (C-N). ^1^H NMR (500 MHz, DMSO-d_6_): δ 13.93 (s, 1H, SH), 9.85 (dd, *J* = 8.2 Hz, 1H), 7.59 (dq, *J* = 7.9 Hz, 2H, ArH), 7.43 (dq, *J* = 7.8 Hz, 2H, ArH), 7.32–7.16 (m, 2H, ArH), 6.72 (tt, *J* = 13.6, 7.1 Hz, 2H, ArH -CH=CH-), 6.25–6.13 (m, 2H, ArH, -CH=CH-), 2.47 (s, 3H, CH_3_). ^13^C NMR (126 MHz, DMSO-d_6_): δ 167.6, 159.0, 149.7, 136.3, 133.7, 129.6, 129.6, 129.0, 128.6, 125.9, 115.8, 107.3, 6.5.
*(E)*-5-(2-(Benzo[d][1,3]dioxol-5-yl)vinyl)-4-phenyl-4H-1,2,4-triazole-3-thiol (**4f**): Yield: 99%, white solid, m.p.: 283–284 °C (recrystallization from C_2_H_5_OH). IR (KBr, cm^−1^): 2701 (S-H), 1633 (N=C), 1293 (C-N). ^1^H NMR (500 MHz, DMSO-d_6_): δ 13.98 (s, 1H, SH), 7.64–7.53 (m, 3H, ArH), 7.46–7.40 (m, 2H, ArH), 7.22 (d, *J* = 16.3 Hz, 1H, -CH=CH-), 7.14 (s, 1H, ArH), 6.91 (dd, *J* = 8.1, 1H, ArH), 6.88 (d, *J* = 8.0 Hz, 1H, ArH), 6.33 (d, *J* = 16.3 Hz, 1H, -CH=CH-), 6.02 (s, 2H, CH_2_). ^13^C NMR (126 MHz, DMSO-d_6_): δ 167.7, 149.5, 148.5, 148.0, 136.1, 133.6, 129.6, 129.3, 128.5, 123.3, 109.2, 108.6, 106.0, 101.5.
*(E)*-5-(4-((4-Bromobenzyl)oxy)styryl)-4-phenyl-4H-1,2,4-triazole-3-thiol (**4g**): Yield: 73%, yellow solid, m.p.: 278–279 °C (recrystallization from C_2_H_5_OH). IR (KBr, cm^−1^): 2659 (S-H), 1603 (N=C), 1545, 1298 (C-N). ^1^H NMR (500 MHz, DMSO-d_6_): δ 13.99 (s, 1H, SH), 7.64–7.54 (m, 5H, ArH), 7.46–7.42 (m, 2H, ArH), 7.39 (dd, *J* = 8.5 Hz, 4H, ArH), 7.27 (d, *J* = 16.3 Hz, 1H, -CH=CH-), 7.00–6.94 (m, 2H, ArH), 6.29 (d, *J* = 16.3 Hz, 1H, -CH=CH-), 5.09 (s, 2H, CH_2_). ^13^C NMR (126 MHz, DMSO-d_6_): δ 167.6, 159.2, 149.5, 136.2, 135.8, 133.6, 131.4, 129.9, 129.6, 128.9, 128.5, 127.8, 121.0, 115.3, 108.7, 68.5.

#### 3.2.4. General Method for the Synthesis of Oxadiazole-Cinnamic Acid Derivatives (**5a**–**5c**)

For the synthesis of the final oxadiazole derivatives, 1-ethyl-3-(3-dimethylaminopropyl)carbodiimide hydrochloride (1 equiv) was added to cinnamoyl-thiosemicarbazide derivatives in dimethyl sulfoxide (DMSO). The reaction was carried out under reflux at 60 °C for approximately 3 h. The solution is then extracted with dichloromethane (DCM) and deionized water. The organic phases are collected, washed with saturated NaCl solution, dried over anhydrous Na_2_SO_4_, and concentrated to obtain the final product.

*(E)*-N-Phenyl-5-styryl-1,3,4-oxadiazol-2-amine (**5a**): The spectral data were in agreement with the literature data [53].
*(E)*-5-(4-Chlorostyryl)-N-phenyl-1,3,4-oxadiazol-2-amine (**5b**): Yield: 91%, solid, m.p.: 244–246 °C. IR (KBr, cm^−1^): 3201.8, 3021.3 (NH), 1628.3 (C=C). ^1^H NMR: (500 MHz, DMSO): δ 10.66 (s, 1H, -NH), 7.73 (d, *J* = 8.2 Hz, 2H, ArH), 7.59 (d, *J* = 8.0 Hz, 2H, ArH), 7.44 (d, *J* = 8.2 Hz, 2H, ArH), 7.34–7.24 (m, 4H, ArH, -CH=CH-), 6.98 (t, *J* = 7.3 Hz, 1H, ArH). ^13^C NMR: (126 MHz, DMSO): δ 159.4, 157.9, 138.5, 134.1, 134, 133.9, 129.2, 129.1, 128.9, 122.00, 117.2, 111.3.
*(E)*-5-(4-Fluorostyryl)-N-phenyl-1,3,4-oxadiazol-2-amine (**5c**): Yield: 78%, solid, m.p.: 213–215 °C. IR (KBr, cm^−1^): 3221.6, 3023.7 (NH), 1701.7, 1685.8 (C=C). ^1^H NMR: (500 MHz, DMSO): δ 10.66 (s, 1H, -NH), 7.82–7.79 (m, 2H, ArH), 7.61–7.59 (m, 2H, ArH), 7.37–7.34 (m, 2H, ArH), 7.28–7.19 (m, 4H, ArH, -CH=CH-), 7.01 (t, *J* = 7.3 Hz, 1H, ArH). ^13^C NMR: (126 MHz, DMSO): δ 159.7, 158.3, 147.9, 139, 134.7, 130.1, 129.5, 125.1, 122.3, 117.5, 116.3, 110.8.

#### 3.2.5. General Method for the Synthesis of Thiadiazole-Cinnamic Acid Derivative (**6a**)

For the synthesis of the final thiadiazole derivative, an appropriate amount of concentrated sulfuric acid (H_2_SO_4_) was added to a round-bottom flask. The corresponding cinnamoyl-thiosemicarbazide derivative was added portion wise to the acid under continuous stirring. The reaction was carried out in an ice bath. After 2–3 h, the reaction mixture was poured into ice, resulting in the precipitation of a solid, which was collected by vacuum filtration and washed thoroughly with water several times. The product was then recrystallized from a methanol/water mixture to afford the pure compound.

*(E)-N*-Phenyl-5-styryl-1,3,4-thiadiazol-2-amine (**6a**): The spectral data were in agreement with the literature data [52].

^1^H-NMR and ^13^C-NMR of the novel synthesized derivatives can be found at the Supplementary Material.

### 3.3. Biological in Vitro Assays

A 10 mM stock solution of the synthesized compounds was prepared in DMSO for the vitro assays. Serial dilutions were carried out to determine the IC_50_ values. Each experiment was conducted six times, with standard deviations remaining below 10% of the mean.

#### 3.3.1. Determination of the Reducing Activity of the Stable Radical DPPH

An in vitro study was conducted as previously described by our group. Free radical scavenging activity was evaluated by measuring absorbance at 517 nm after 20 and 60 min at room temperature. NDGA served as the reference compound [59,68]. The results are presented in Table 2.

#### 3.3.2. Inhibition of AAPH-Induced Linoleic Acid Peroxidation

AAPH was employed as an inducer of alkyl peroxyl radicals. In this experiment, the compounds’ ability to inhibit the oxidation of linoleic acid by alkyl peroxyl radicals was assessed by monitoring the change in absorbance at 234 nm due to the formation of 13-hydroperoxy-linoleic acid. Trolox was used as the reference compound [59,68]. The results are presented in Table 2.

#### 3.3.3. Inhibition of Soybean Lipoxygenase

Sodium linoleate (0.1 mM), 0.2 mL of soybean lipoxygenase solution (1/9 × 10^−4^ *w/v* in saline), and 10 μL of the test compound stock solution (10 mM in DMSO) were incubated at room temperature. Absorbance was recorded at 234 nm while monitoring the formation of 13-hydroperoxy-linoleic acid. NDGA was used as the reference compound [59,66,68]. To determine IC_50_ values, various concentrations of the test compounds were applied. The results are presented in Table 2.

### 3.4. Computational Methods

#### 3.4.1. Molecular Docking Studies on Soybean Lipoxygenase

The protein structure (PDB ID: 3PZW) was visualized and preprocessed using UCSF Chimera (version 1.17) [69]. Water molecules and non-essential crystallographic components were removed via Chimera. Missing residues (Met1–Phe2–Ser3–Ala4–Gly5; Glu21–Val22–Asn23–Pro24–Asp25–Gly26–Ser27–Ala28–Val28–Asp29; Ile117–Ser118–Asn119–Gln120) were added using Modeller (v. 10.3) [70]. Hydrogen atoms and partial charges were incorporated using AmberTools 23 [71,72]. The iron center was assigned a +2.0 charge based on the 12–6 Lennard-Jones (LJ) non-bonded model [73]. Histidine residues (His499, His504, His690), which coordinate the iron, were modeled as neutral with δ-nitrogen protonation. The TIP3P water model was used for solvation, with the simulation box maintaining at least 12 Å between the solute and the box boundary. Ligand 3D structures were generated and minimized using Open Babel (v. 3.1.1) [74] with the MMFF94 force field [75]. Ligand topologies and parameters were generated with ACPYPE [76], employing AnteChamber (AmberTools v. 22.10) [77]. Protein energy minimization was executed using GROMACS (v. 4.6.5) [78]. Ligand docking was carried out with AutoDock Vina (v. 1.2.3) [79], using a grid box centered at x = 1.35 Å, y = 14.3 Å, z = −34.60 Å, and with dimensions x = 100 Å, y = 70 Å, z = 70 Å. The exhaustiveness was set to 10, with up to 20 docking modes generated. Docking outcomes were examined using UCSF Chimera.

#### 3.4.2. In Silico Determination of Drug-likeness and Lipophilicity

The compounds’ drugability was performed used the online software Molinspiration (https://www.molinspiration.com/).

Lipophilicity was theoretically calculated as clog *P* values by the Bio-Loom program of BioByte Corp (http://biobyte.com/bb/prod/bioloom.html, accessed on 1 April 2025) [80].

## 4. Conclusions

In conclusion, a total of 18 compounds were synthesized: 7 cinnamoyl-thiosemicarbazide derivatives, 7 triazole derivatives, 3 oxadiazole derivatives, and 1 thiadiazole derivative. Compounds of series 3-5 are new except for derivatives **3a**, **4d**, **5a**, and **6a** which have been previously reported in the literature [52,53,54,55]. From the literature, compound **4d** has been proved to be an allosteric Valosine-containing protein inhibitor [54]. All the synthesized compounds were evaluated for their antioxidant ability to (a) interact with the free radical DPPH, (b) inhibit lipid peroxidation, and (c) inhibit soybean lipoxygenase. The structures of the compounds were identified by melting point measurement and spectroscopic techniques (IR, ^1^H NMR, ^13^C NMR). All the cinnamoyl-thiosemicarbazide derivatives (**3a, 3b**, **3c**, **3d**, **3f**, and **3g**) demonstrated strong DPPH radical scavenging activity after 60 min with the exception of compound **3e**. Compound **3d** exhibited the highest activity at 97%. Among the triazole derivatives, compounds **4e** and **4g** exhibit the highest DPPH scavenging activity. It seems that cyclization to the hybrid cinnamoyl-triazole structure positively contributed to the interaction with the DPPH radical at 60 min, except for compounds **3d**, **4d**, and **3f**, **4f**. Also, the increase in lipophilicity, clog *P* (**3a**) < clog *P* (**4a**) < clog *P* (**6a**), seems to enhance the interaction with the stable free radical DPPH. Based on the IC_50_ values, the triazole derivatives exhibit a stronger inhibitory effect against soybean lipoxygenase compared to the cinnamoyl-thiosemicarbazide derivatives. For triazole derivatives, compounds **4b** and **4g** possess the highest inhibitory effect against sLOX. Cyclization of compounds **3a**–**3g** to **4a**–**4g** seems to contribute positively to the anti-LOX activity with the exception of compounds **3b** and **3c**. This is followed also by compounds **5a**–**c** with the exception of compound **5a**. All the derivatives have been subjected to docking studies and they seem to bind in a similar mode to the enzyme. The design through docking studies highlighted that cyclization enhances the biological activity with the exception of compound **3g**. Compound **4g**, with the most potent anti-LOX activity, develops hydrophobic interactions with amino acids Val126, Tyr525, Lys526, Arg533, Trp772. These findings combined with additional computational studies can be utilized in the design of novel five-membered heterocycle-cinnamic acid derivatives possessing dual antioxidant and anti-lipoxygenase activities.

## Figures and Tables

**Table 1:** Drug-likeness studies of synthesized compounds.

Compound	Milog P ^a^	TPSA ^b^	No. of Atoms	No. of O and N ^c^	No. of OH and NH ^d^	No. of Violations	No. of Rotational Bonds ^e^	Volume ^f^	MW ^g^	Clog *P* ^h^
**3a**	2.78	53.15	21	4	3	0	6	266.92	297.38	2.39
**3b**	3.46	53.15	22	4	3	0	6	280.46	331.83	3.10
**3c**	2.94	53.15	22	4	3	0	6	271.85	315.37	2.53
**3d**	3.59	53.15	22	4	3	0	6	284.81	376.28	3.25
**3e**	2.33	79.46	25	6	3	0	8	311.45	355.42	1.74
**3f**	2.67	71.62	24	6	3	0	6	290.85	341.39	2.36
**3g**	5.24	62.39	30	5	3	1	9	382.00	482.40	4.94
**4a**	3.93	30.72	20	3	0	0	3	248.99	279.37	4.53
**4b**	4.61	30.72	21	3	0	0	3	262.53	313.81	5.24
**4c**	4.09	30.72	21	3	0	0	3	253.92	297.36	5.39
**4d**	4.74	30.72	21	3	0	0	3	266.88	358.26	4.67
**4e**	3.48	57.02	24	5	0	0	5	293.52	337.40	3.88
**4f**	3.82	49.19	23	5	0	0	3	272.92	323.28	4.5
**4g**	6.39	39.95	29	4	0	1	6	364.07	464.39	7.08
**5a**	3.94	50.95	20	4	1	0	4	239.94	263.30	3.93
**5b**	4.61	50.95	21	4	1	0	4	253.47	297.75	4.64
**5c**	4.10	50.95	21	4	1	0	4	244.87	281.29	4.08
**6a**	4.76	37.81	20	3	1	0	4	249.08	279.37	4.76

^a^ Logarithm of partition coefficient between n-octanol and water (milog P); ^b^ topological polar surface area (TPSA); ^c^ number of hydrogen bond acceptors; ^d^ number of hydrogen bond donors; ^e^ number of rotatable bonds; ^f^ molecular volume; ^g^ molecular weight; ^h^ theoretically calculated lipophilicity.

**Table 2:** Interaction with DPPH at 20 and 60 min, inhibition of AAPH-induced linoleic acid peroxidation (AAPH%), and in vitro inhibitory activity against soybean lipoxygenase (expressed as % inhibition and IC_50_) were evaluated for the synthesized compounds.

Compound	RA % 100 μM 20 min	RA % 100 μM 60 min	AAPH % Inhibition 100 μM	LOX % Inhibition 100 μM or IC_50_ (μM)
**3a**	61	92	67	8.5 μM
**3b**	90	83	37	49%
**3c**	90	91	12	45%
**3d**	29	97	33	8 μM
**3e**	83	21	47	9.75 μM
**3f**	70	89	58	35 μΜ
**3g**	98	87	91	10 μM
**4a**	74	93	26	7.5 μM
**4b**	33	88	34	4.5 μM
**4c**	82	93	94	35%
**4d**	68	57	20	97.5 μM
**4e**	96	93	87	5.5 μM
**4f**	87	76	71	7.5 μM
**4g**	97	92	50	4.5 μM
**5a**	n.a.	7	17	32.5 μM
**5b**	65	72	n.a.	18 μM
**5c**	92	82	63	17 μM
**6a**	98	96	60	5 μM
**NDGA**	87	93	-	93 (0.45 μM)
**Trolox**	-	-	92	-

n.a.: no activity under the experimental conditions. Standard deviation (±SD) of the mean is less than < 10%. For the in vitro assays, the mean value is derived from at least six independent experiments.

## Data Availability

The original contributions presented in this study are included in the article. Further inquiries can be directed to the corresponding authors.

## Supplementary Materials

The following supporting information can be downloaded at: https://www.mdpi.com/article/10.3390/molecules30153148/s1. ^1^H NMR and ^13^C NMR of the novel synthesized derivatives.
