# Supplementary material for: Synthesis and Molecular Modeling of Antioxidant and Anti-Inflammatory Five-Membered Heterocycle–Cinnamic Acid Hybrids
Source: Molecules. 2025 Jul 27;30(15):3148. doi: 10.3390/molecules30153148 (PMC12348798; doi:10.3390/molecules30153148)
Supplement: Supplementary file 1 [file molecules-30-03148-s001.zip › molecules-3754148-supplementary.pdf]

Supporting information about the general structures, substitution, yields and references of the novel derivatives

**Table S1.** Cinnamoyl-thiosemicarbazide derivatives (**3a-g**), triazol cinnamic acid derivatives (**4a-g**), oxadiazole cinnamic acid derivatives (**5a-c**) and thiadiazole cinnamic acid derivative (**6a**).

| **Compd** | **General Structure** | **R** | **A%** | **Reference** |
|---|---|---|---|---|
| **3a** | 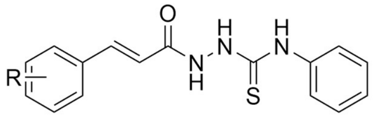 | 4-H | 80 | [55] |
| **3b** | | 4-Cl | 73 | - |
| **3c** | | 4-F | 45 | - |
| **3d** | | 4-Br | 79 | - |
| **3e** | | 4-$CH_3COO$- | 46 | - |
| **3f** | | 3,4-$CH_2OCH_2$- | 45 | - |
| **3g** | | 4-Br-Ph-$CH_2$-O- | 78 | - |
| **4a** | 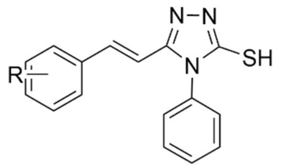 | 4-H | 89 | - |
| **4b** | | 4-Cl | 99 | - |
| **4c** | | 4-F | 99 | - |
| **4d** | | 4-Br | 88 | [54] |
| **4e** | | 4-$CH_3COO$- | 91 | - |
| **4f** | | 3,4-$CH_2OCH_2$- | 99 | - |
| **4g** | | 4-Br-Ph-$CH_2$-O- | 73 | - |
| **5a** | 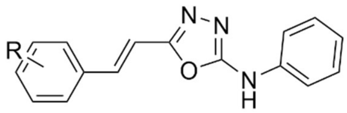 | 4-H | 69 | [53] |
| **5b** | | 4-Cl | 91 | - |
| **5c** | | 4-F | 78 | - |
| **6a** | 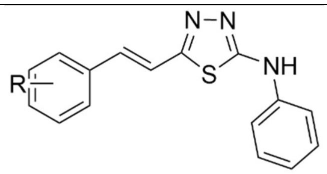 | 4-H | 57 | [52] |

Supporting information about the $^{1}$H-NMR and $^{13}$C-NMR spectra of synthesized derivatives

**4Cl_PITC** corresponds to compound **3b**

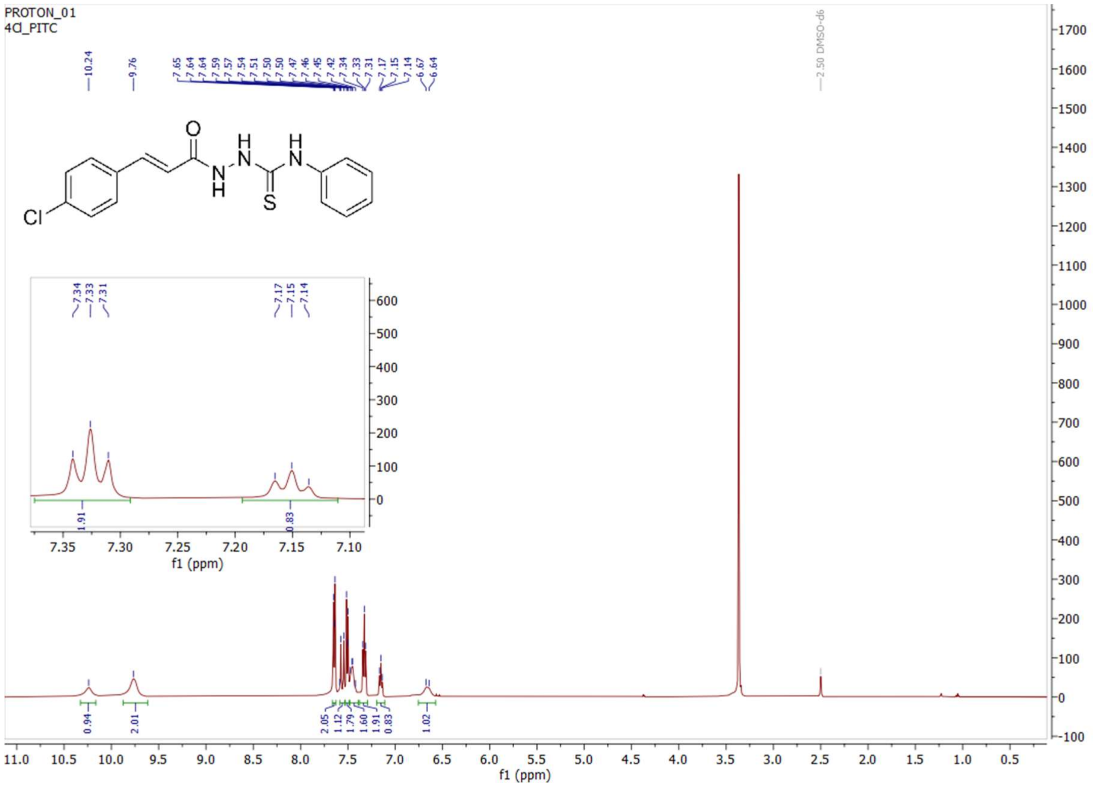

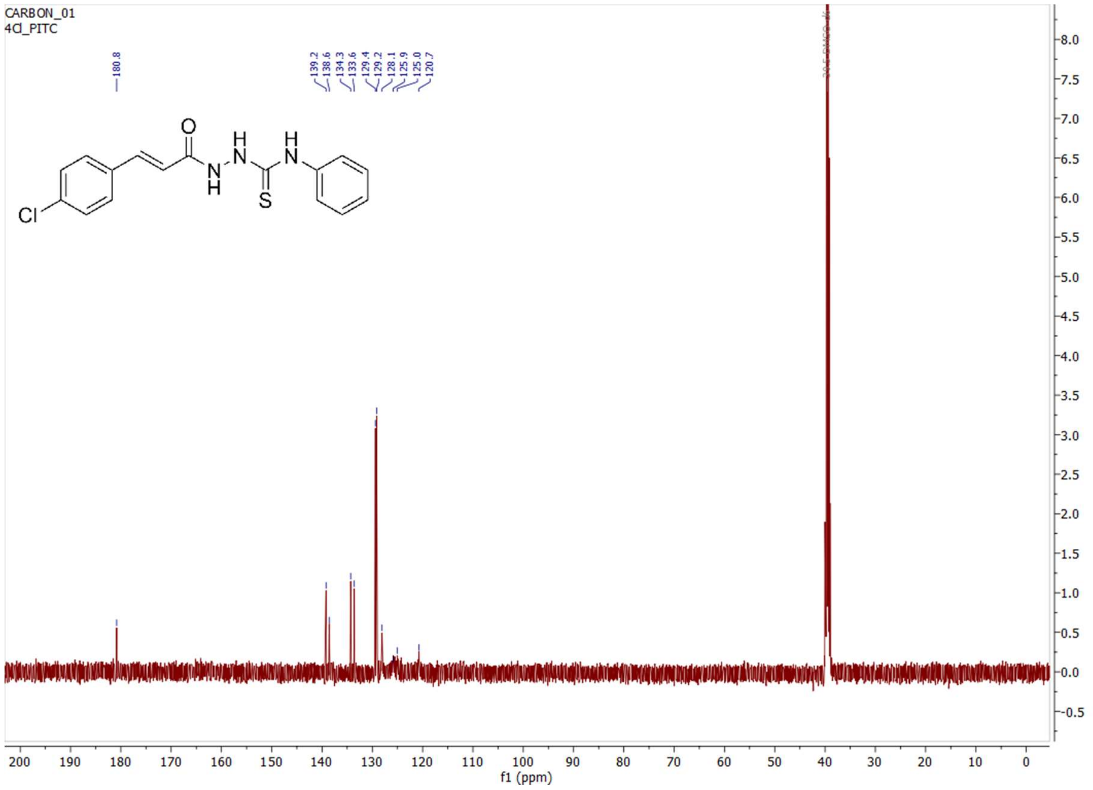

**pF_PITC** corresponds to compound **3c**

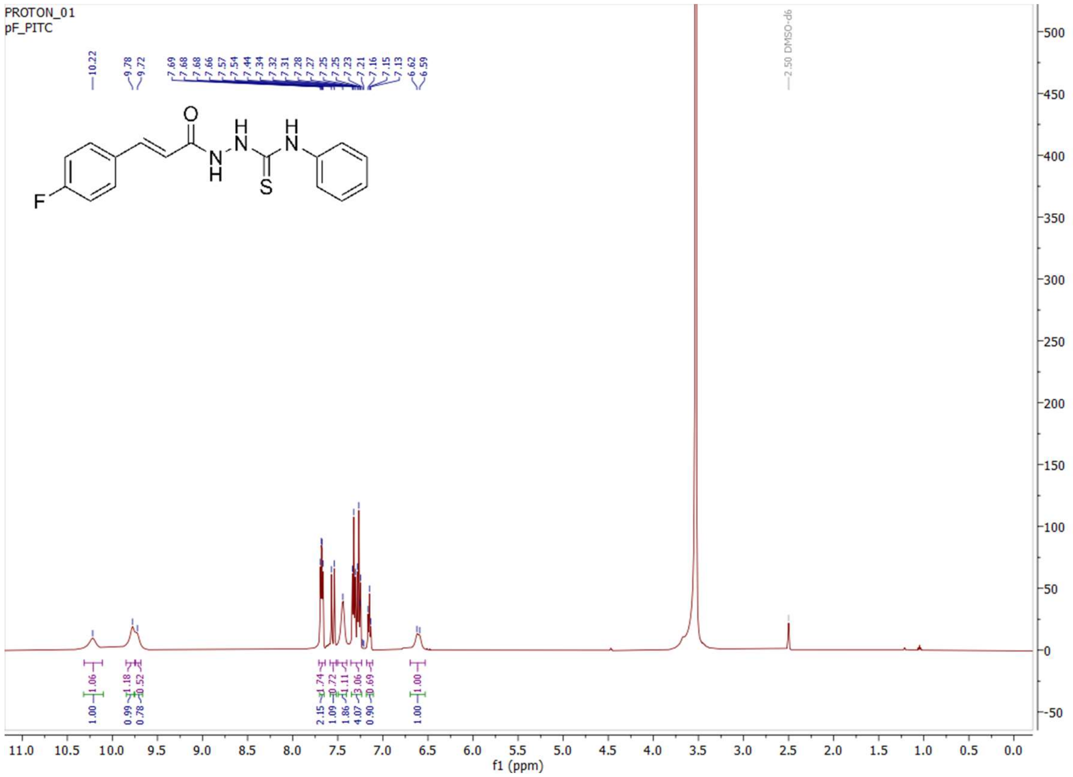


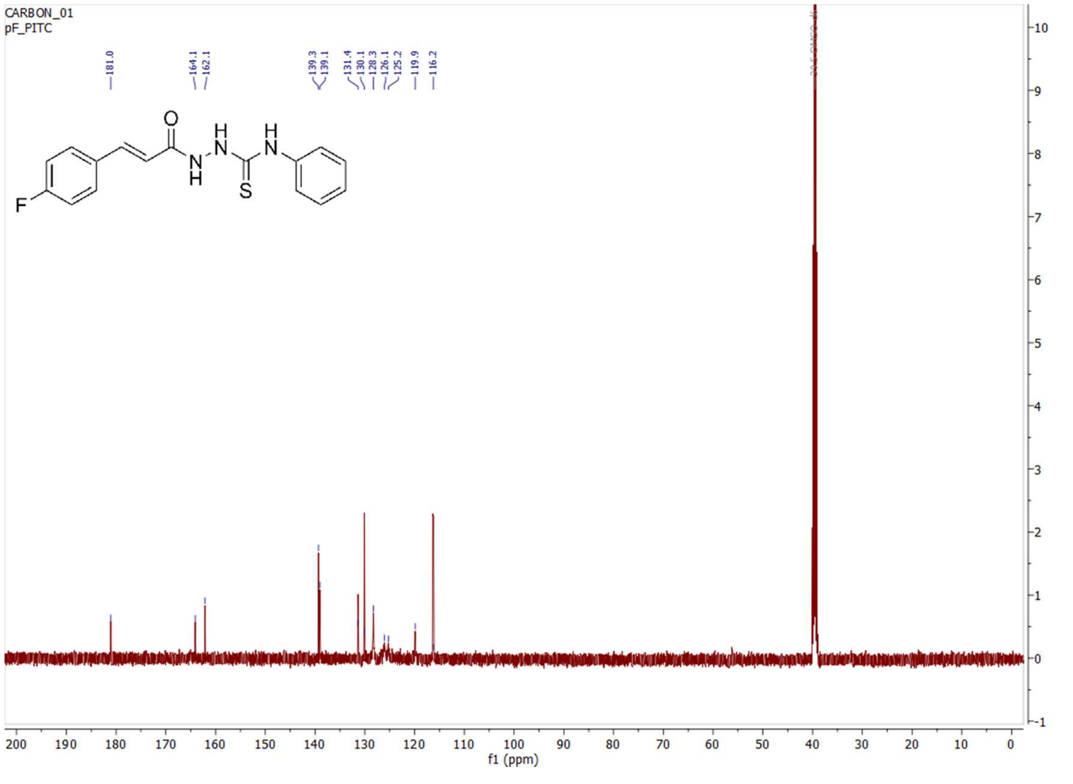

**4Br_PITC** corresponds to compound **3d**

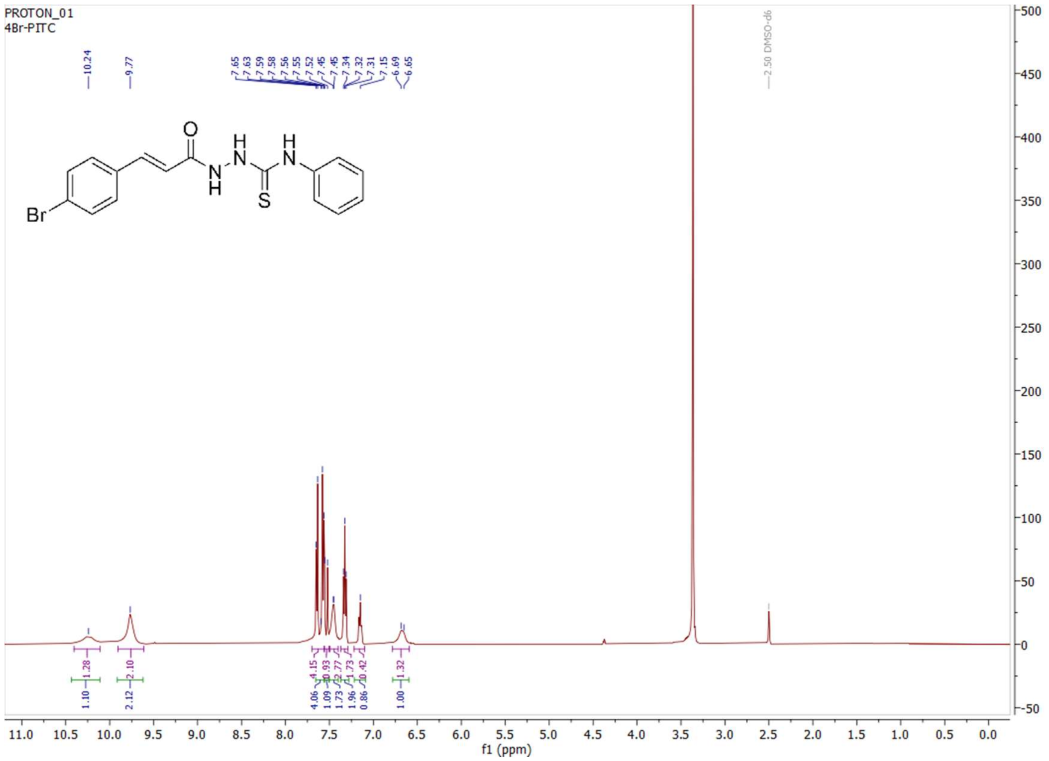


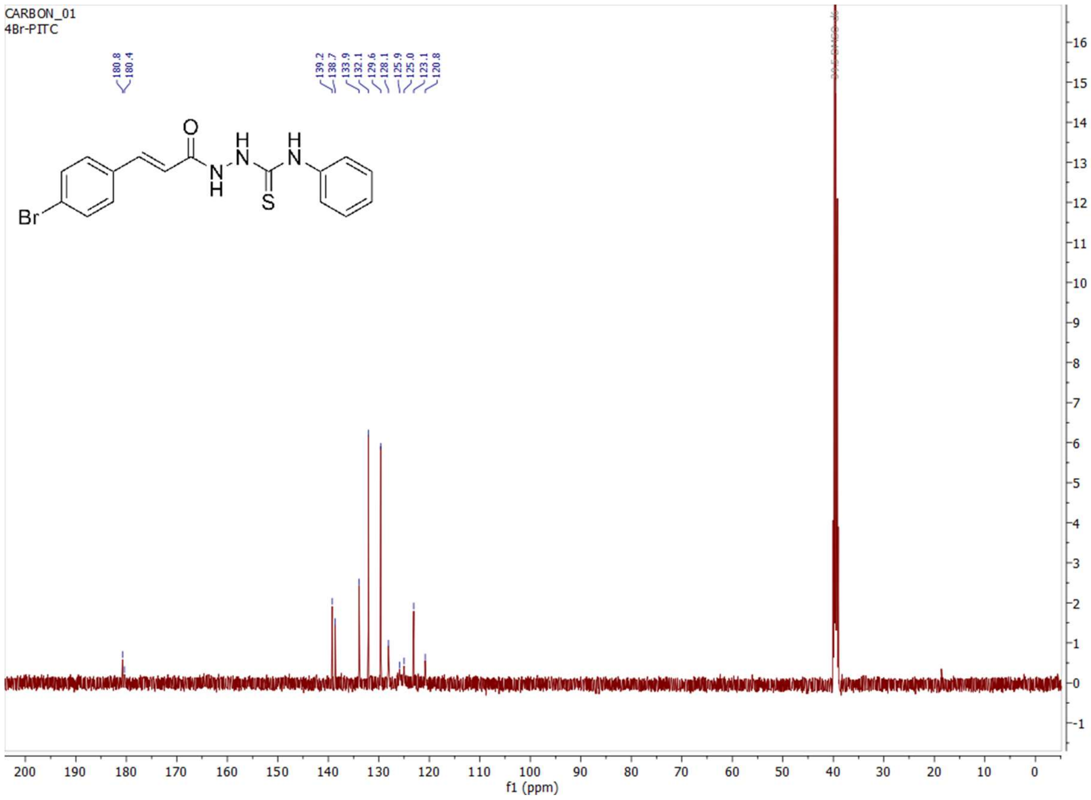

**TK48** corresponds to compound **3e**

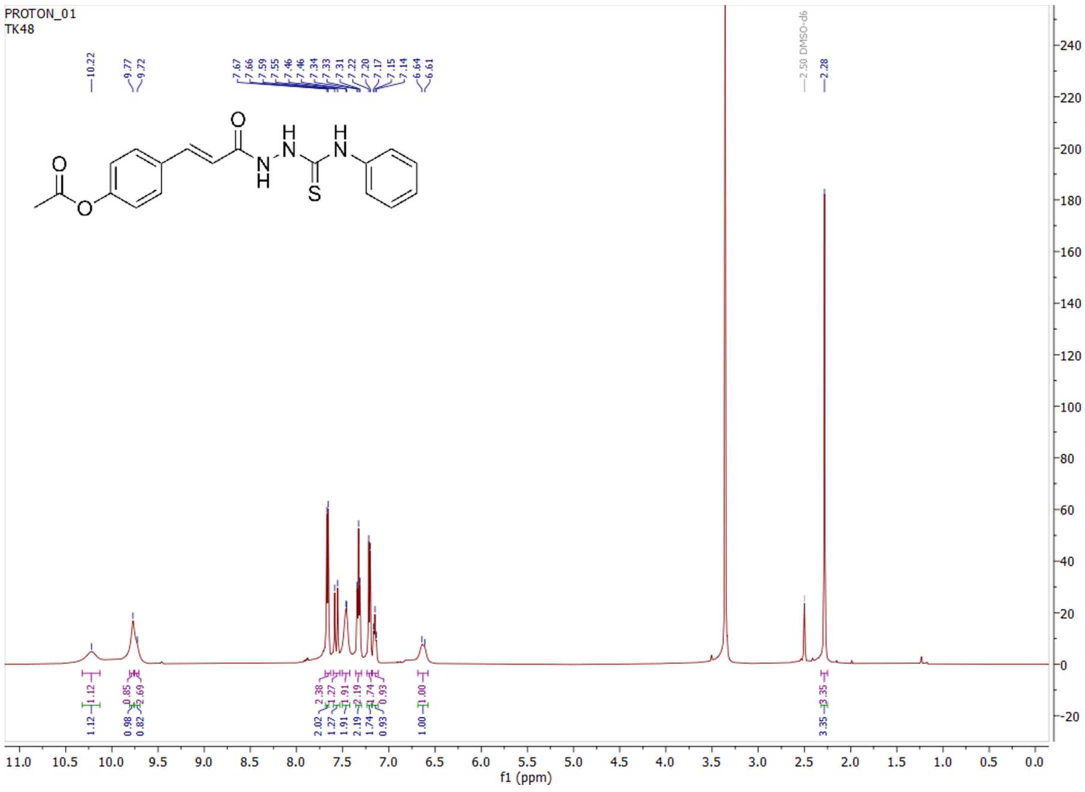


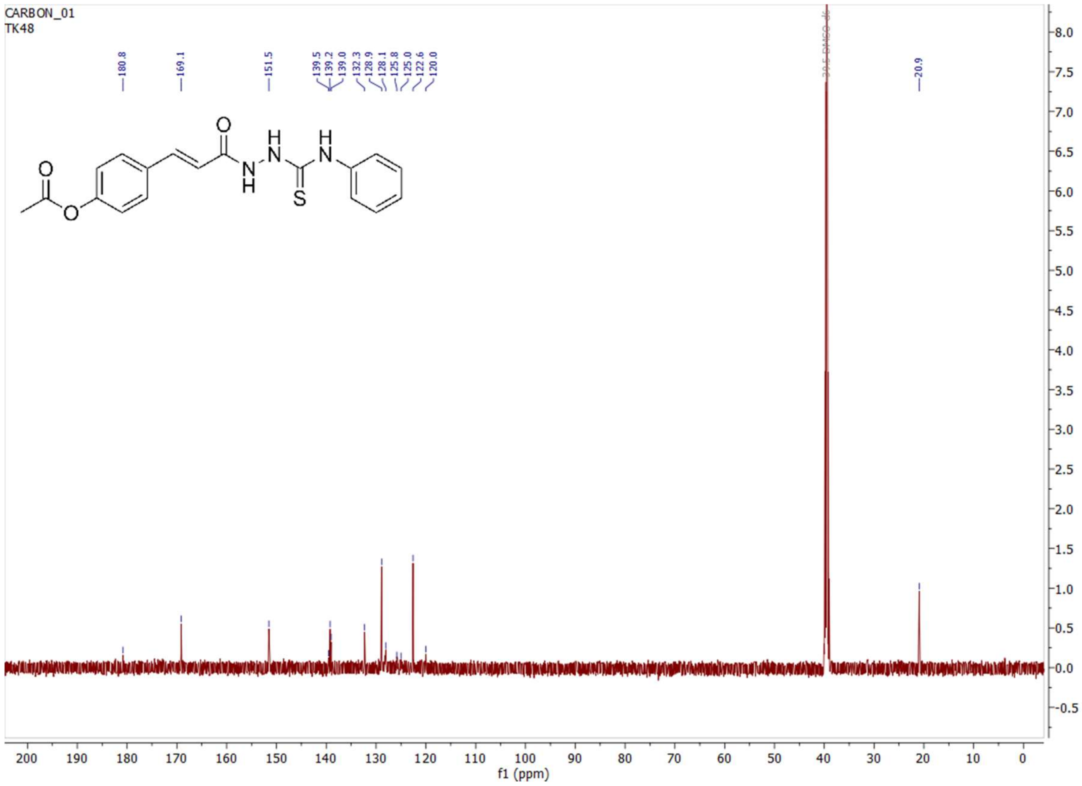

**TK50ag** corresponds to compound **3f**

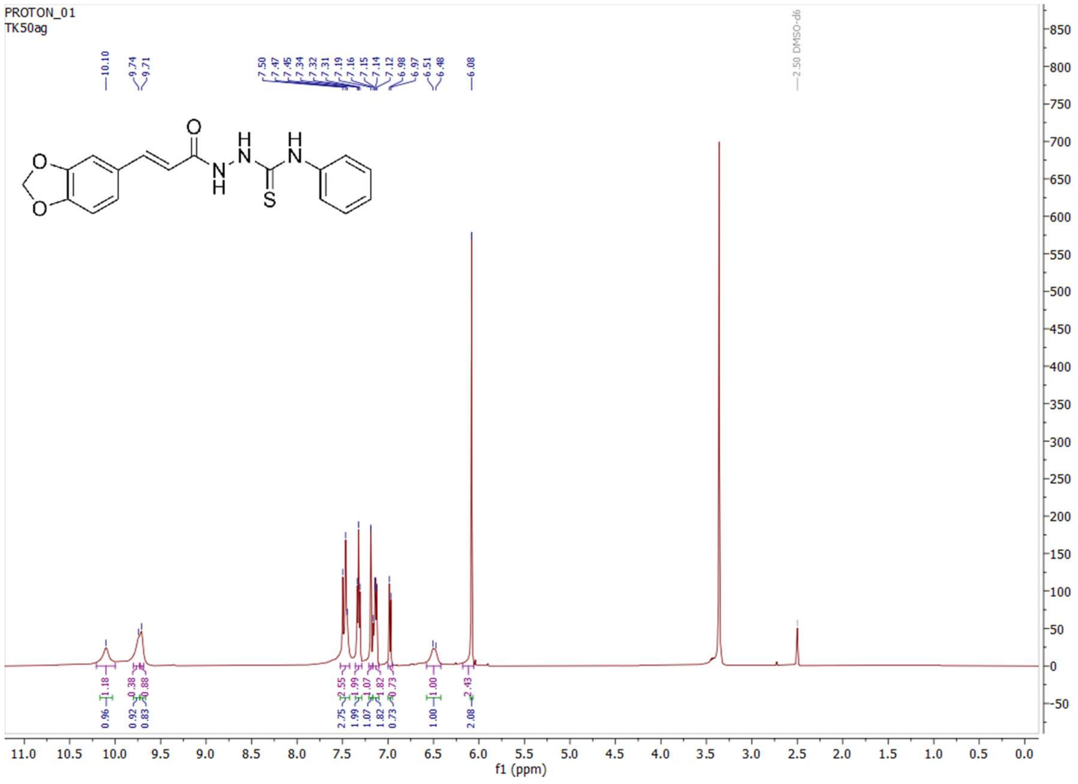


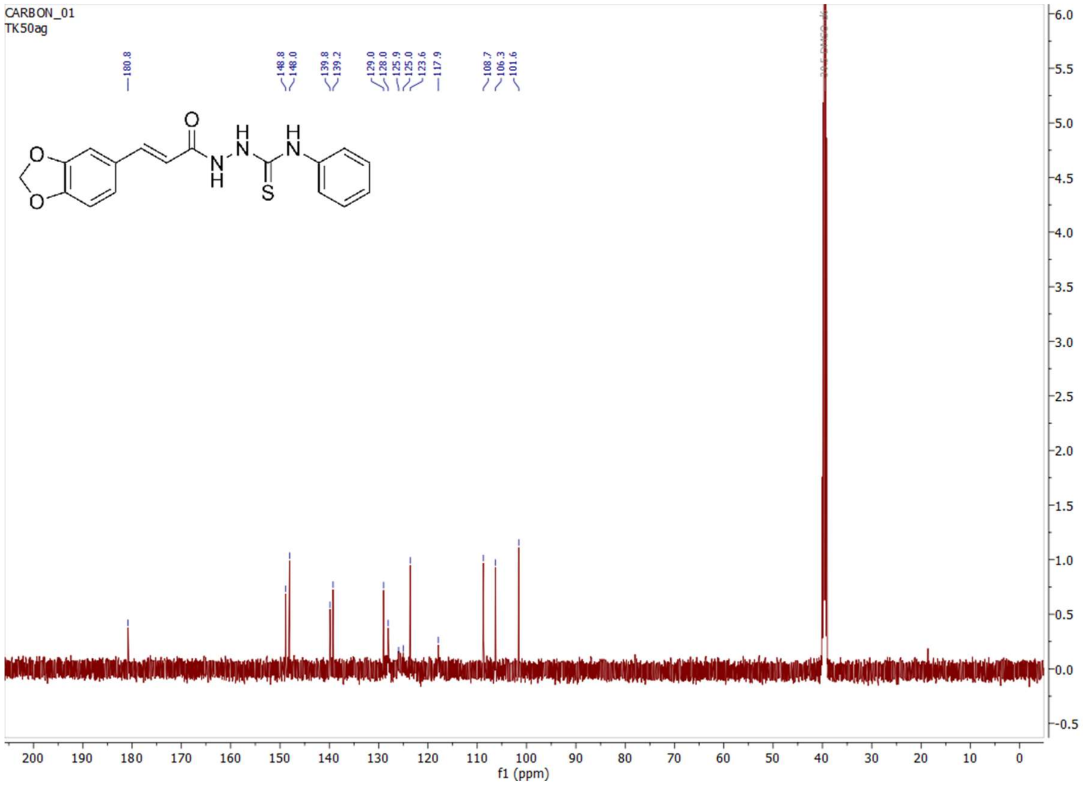

**TK49ag** corresponds to compound **3g**

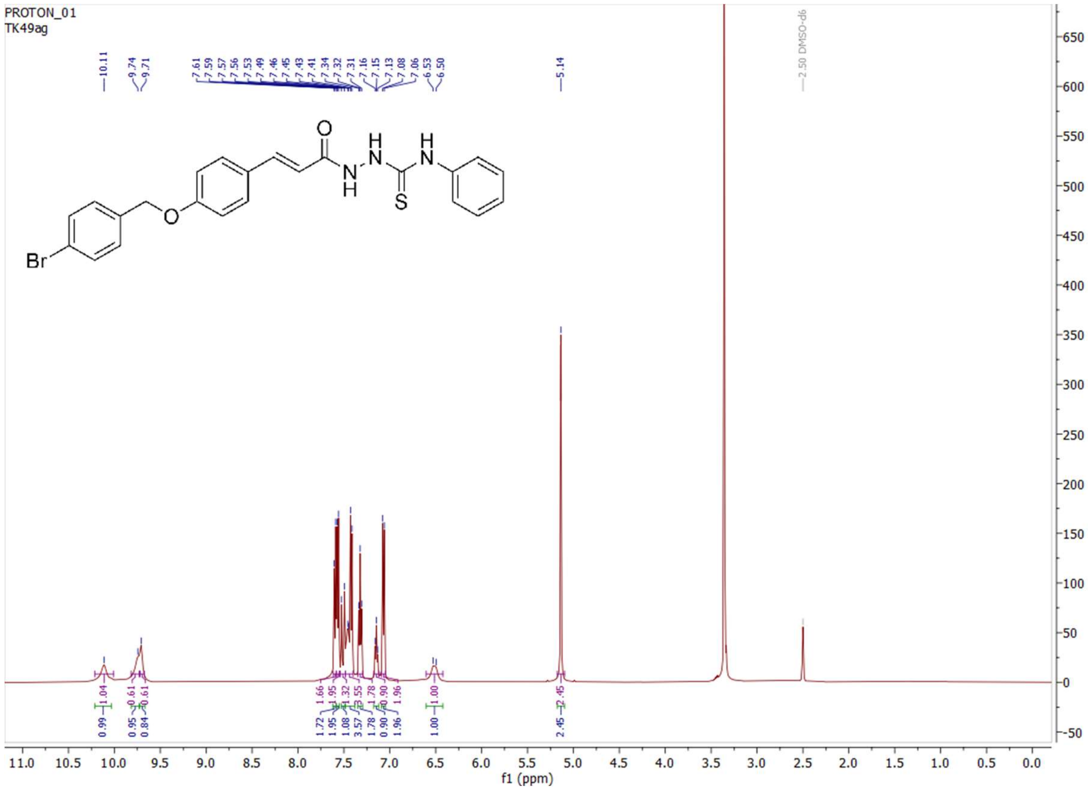


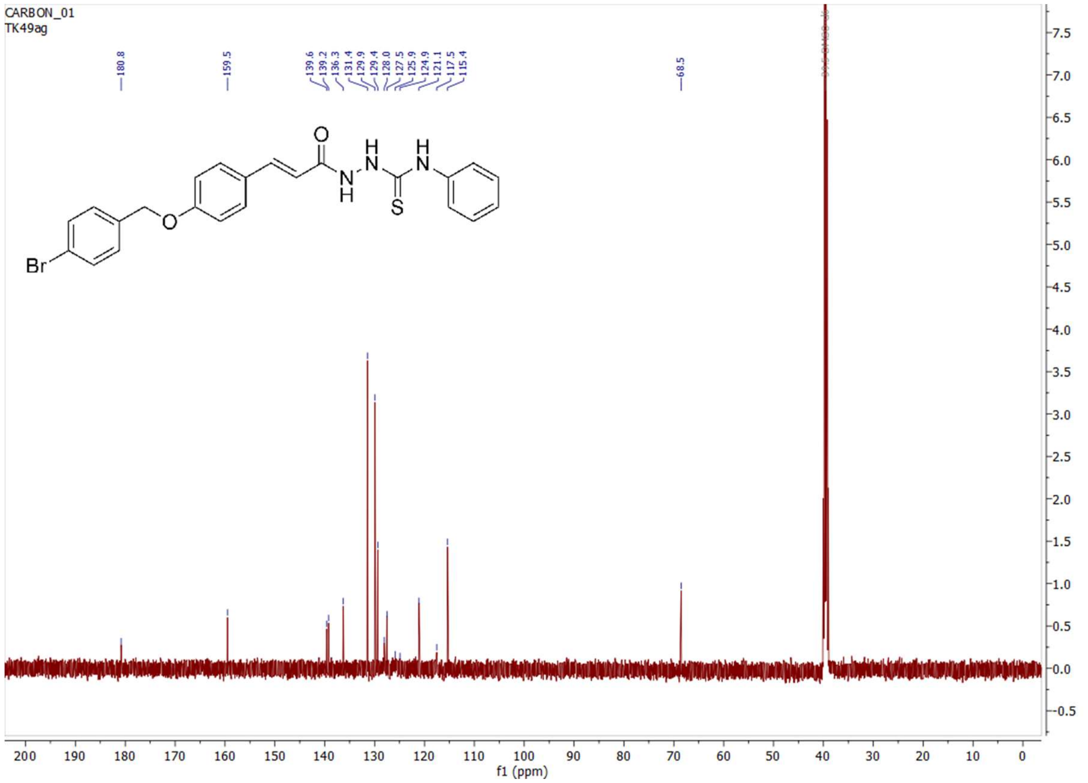

**TK25** corresponds to compound **4a**

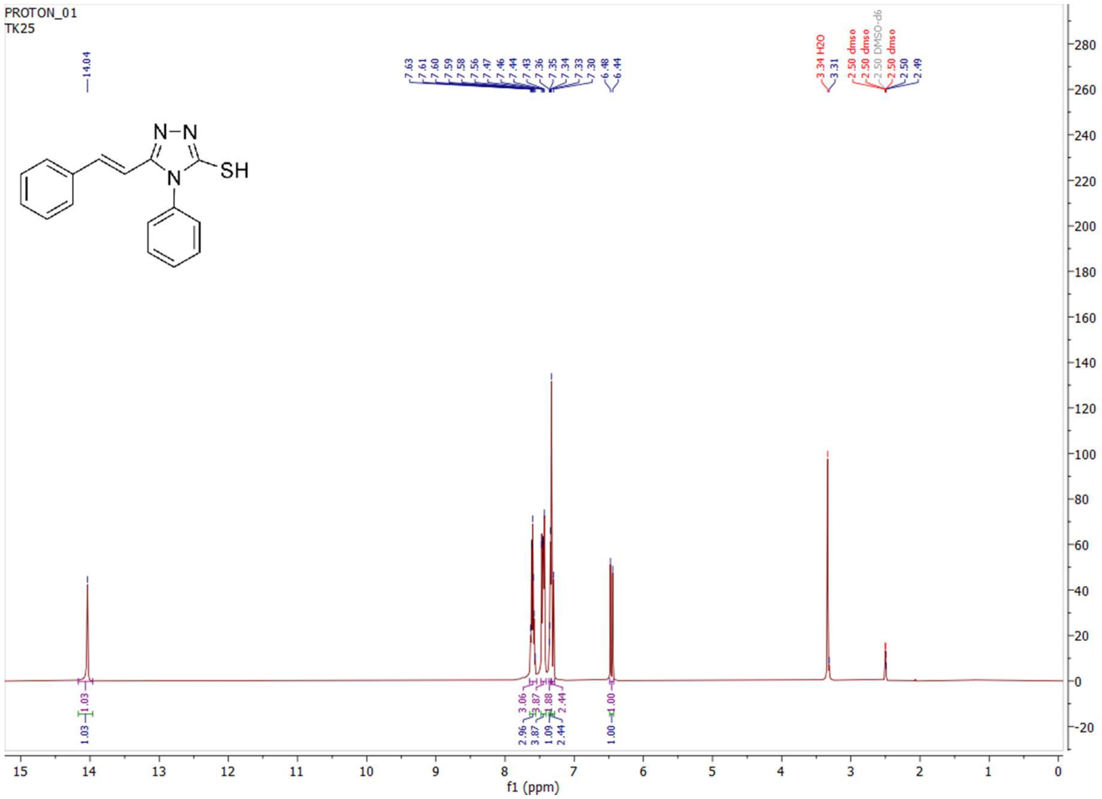


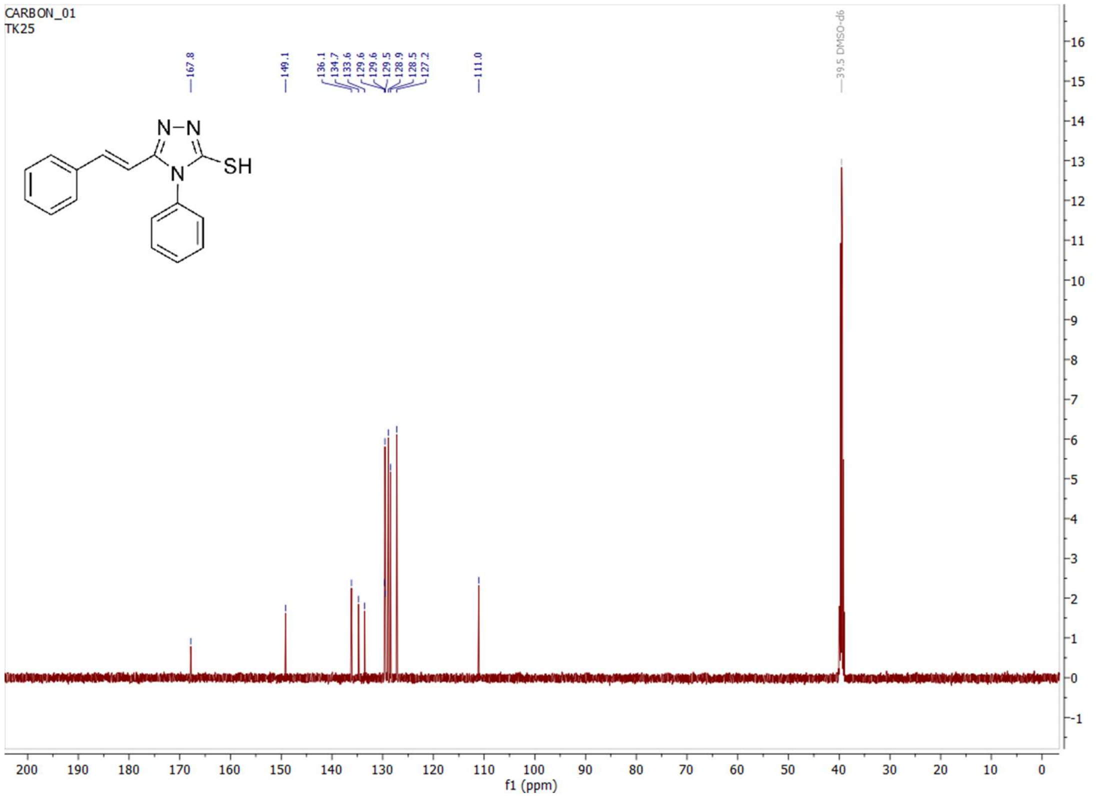

**TK34** corresponds to compound **4b**

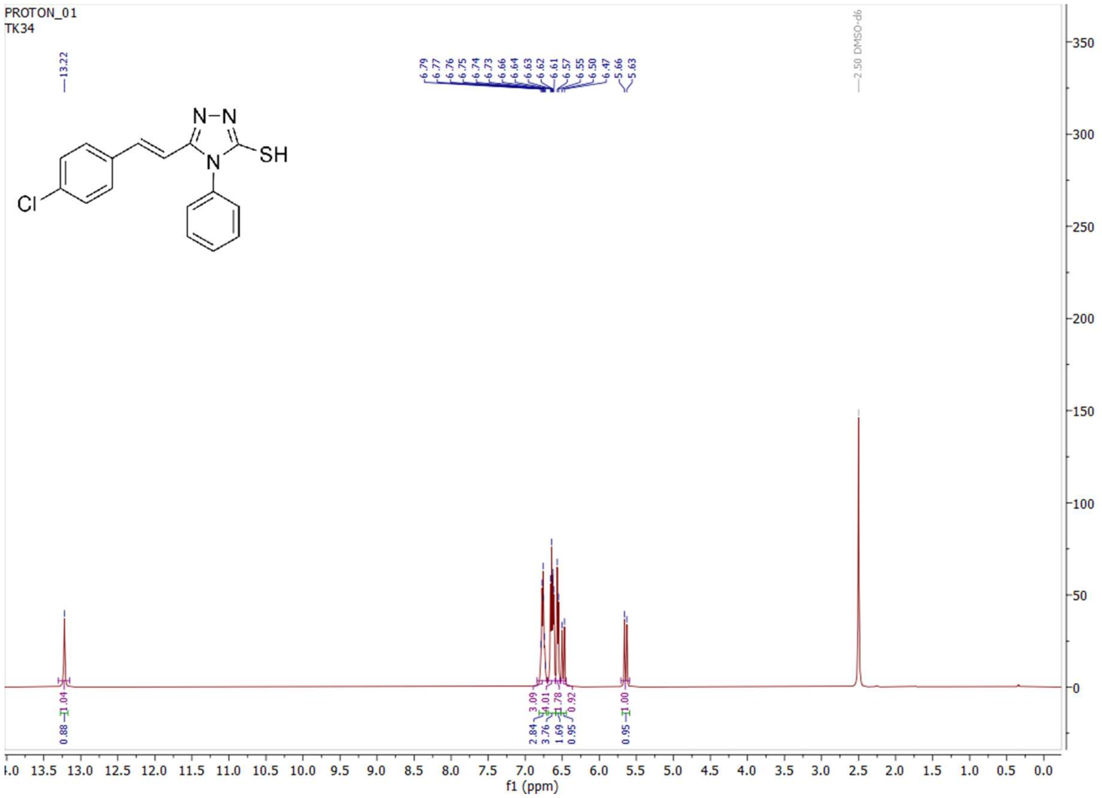


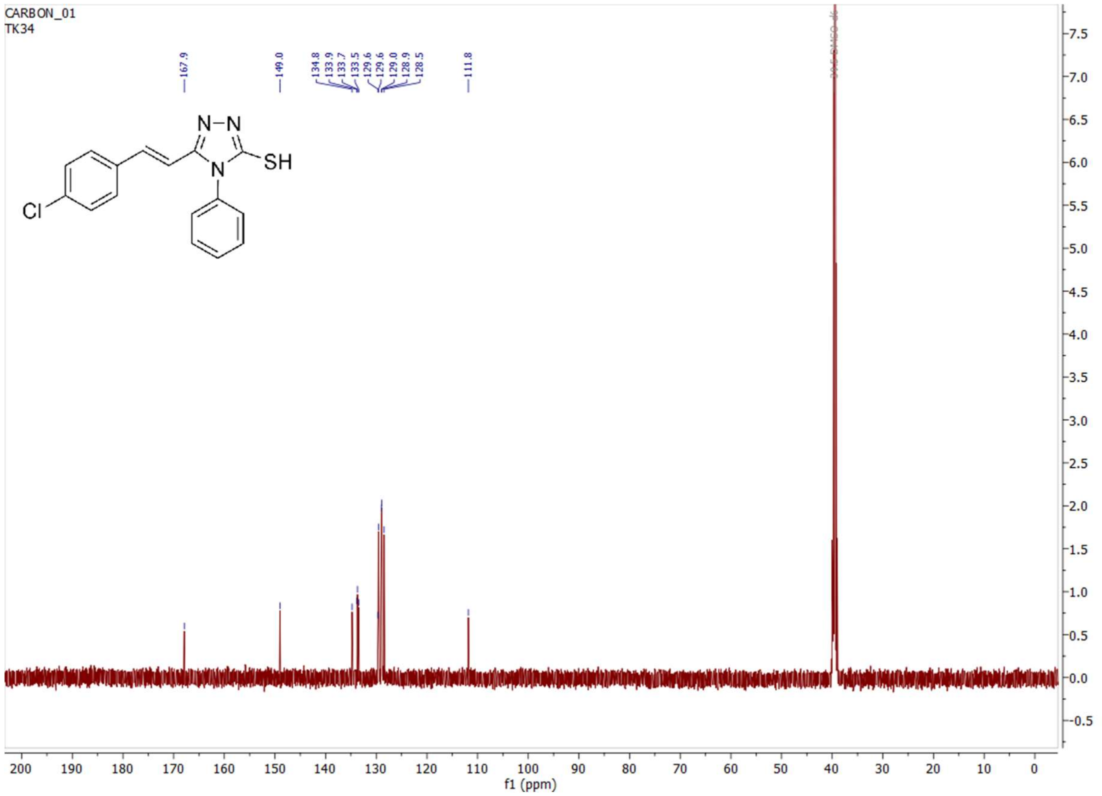

**pF_triazol** corresponds to compound **4c**

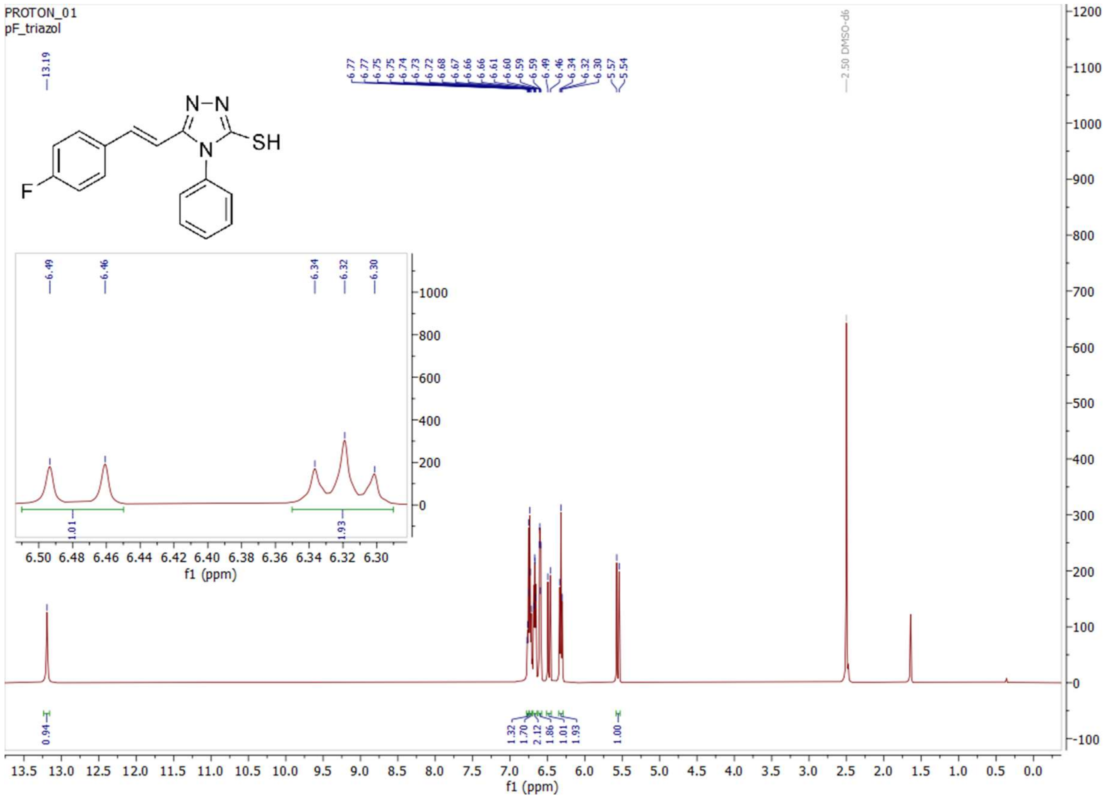


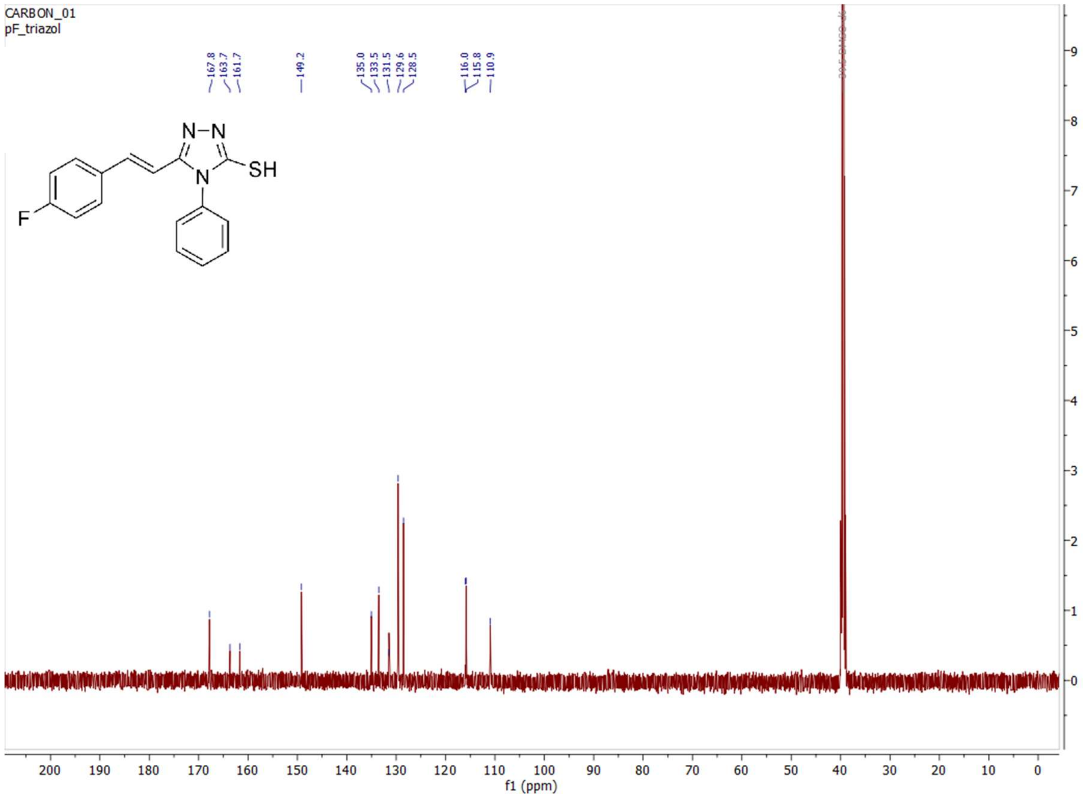

**CH₃COOtriazol** corresponds to compound **4e**

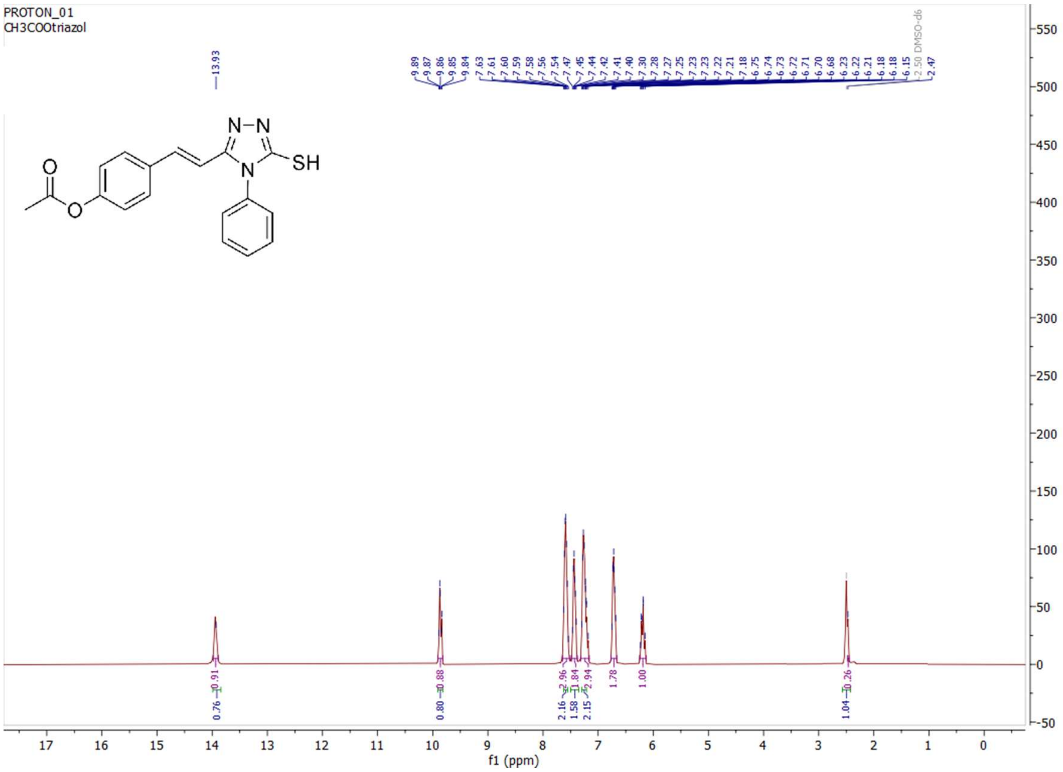


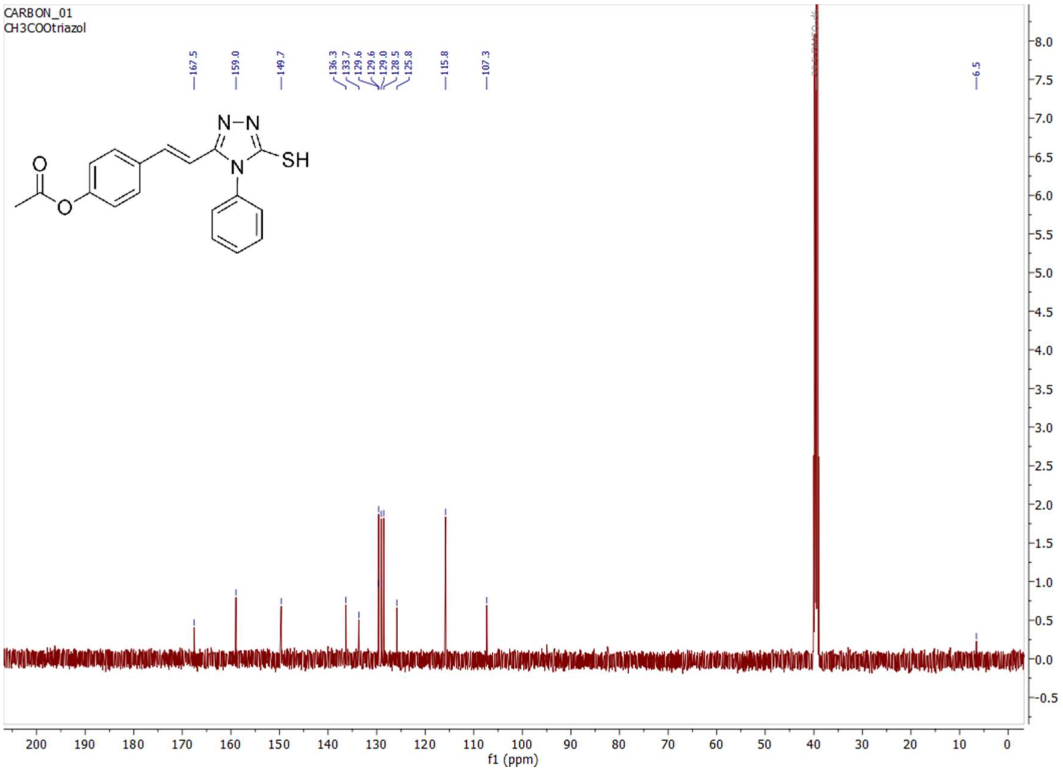

**Dimethoxytriazol** corresponds to compound **4f**

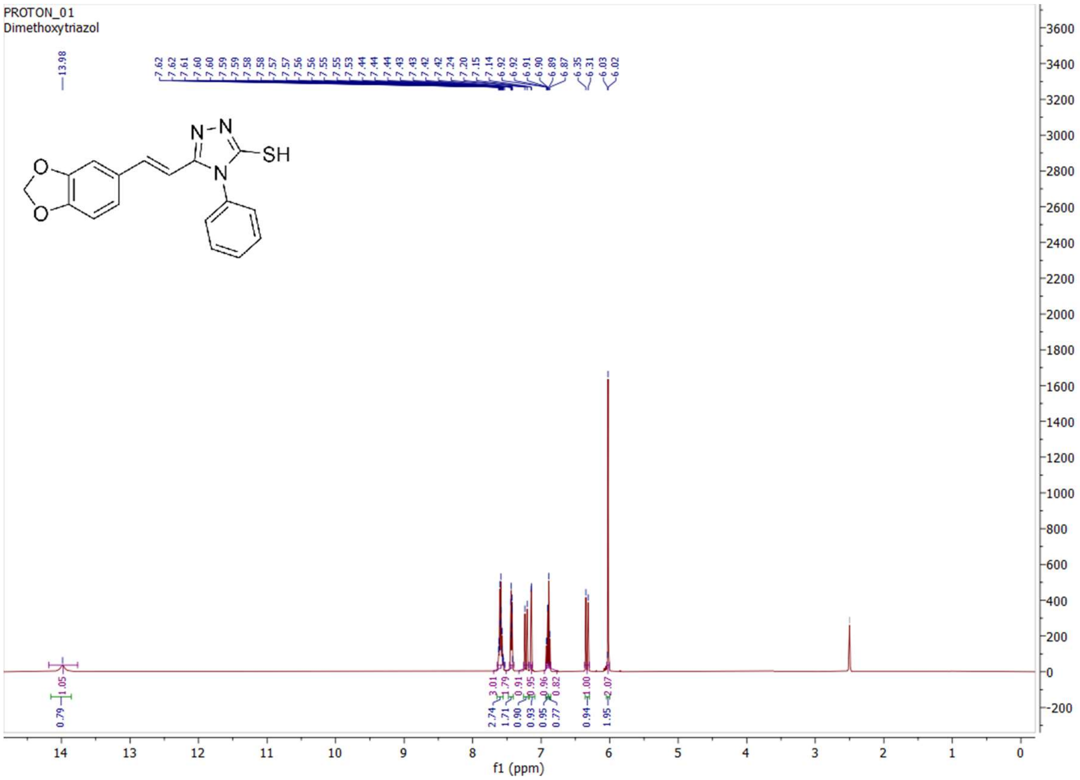


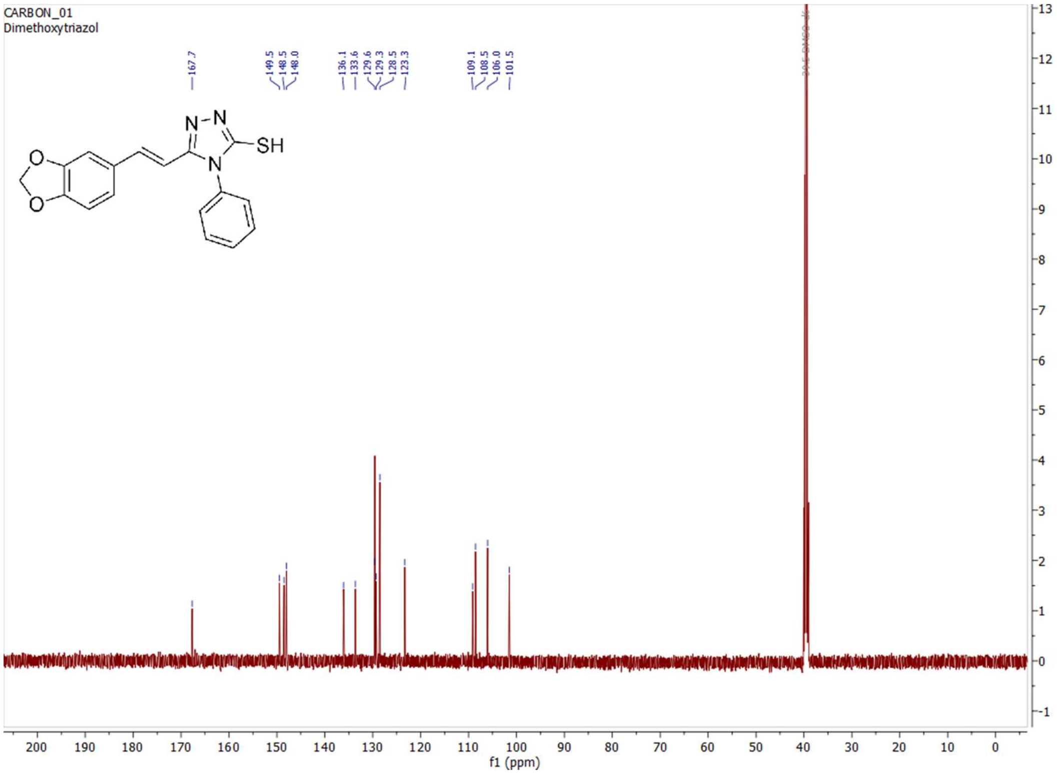

**BrBO_triazol** corresponds to compound **4g**

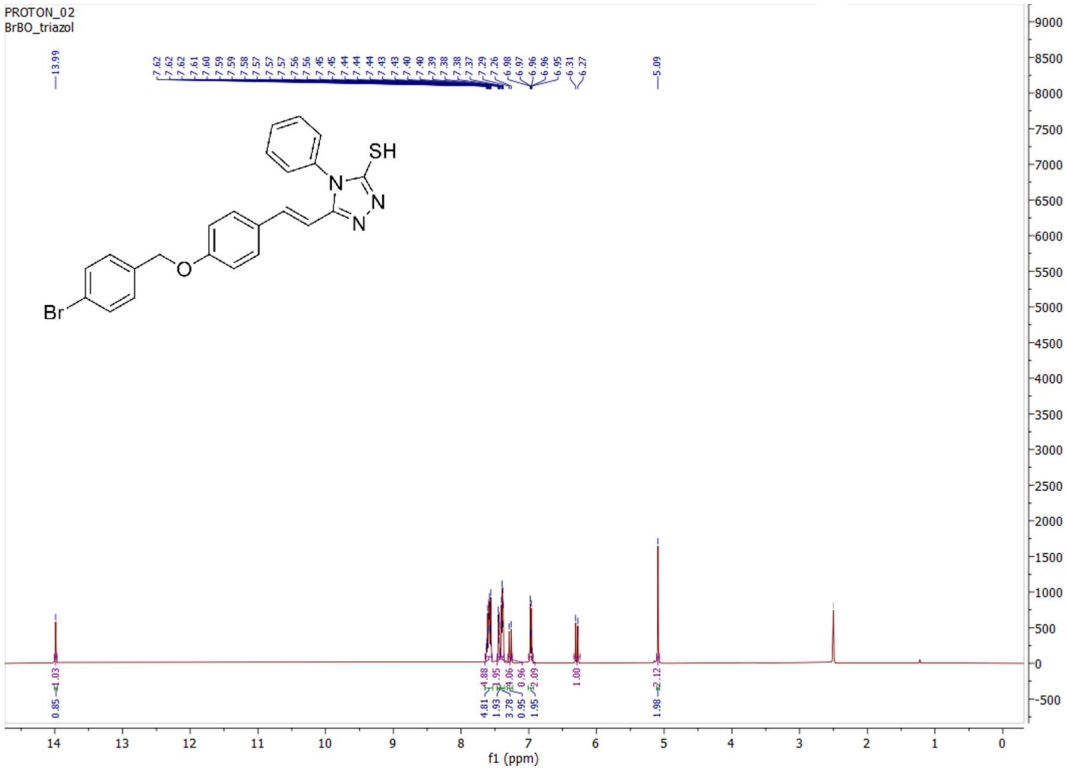


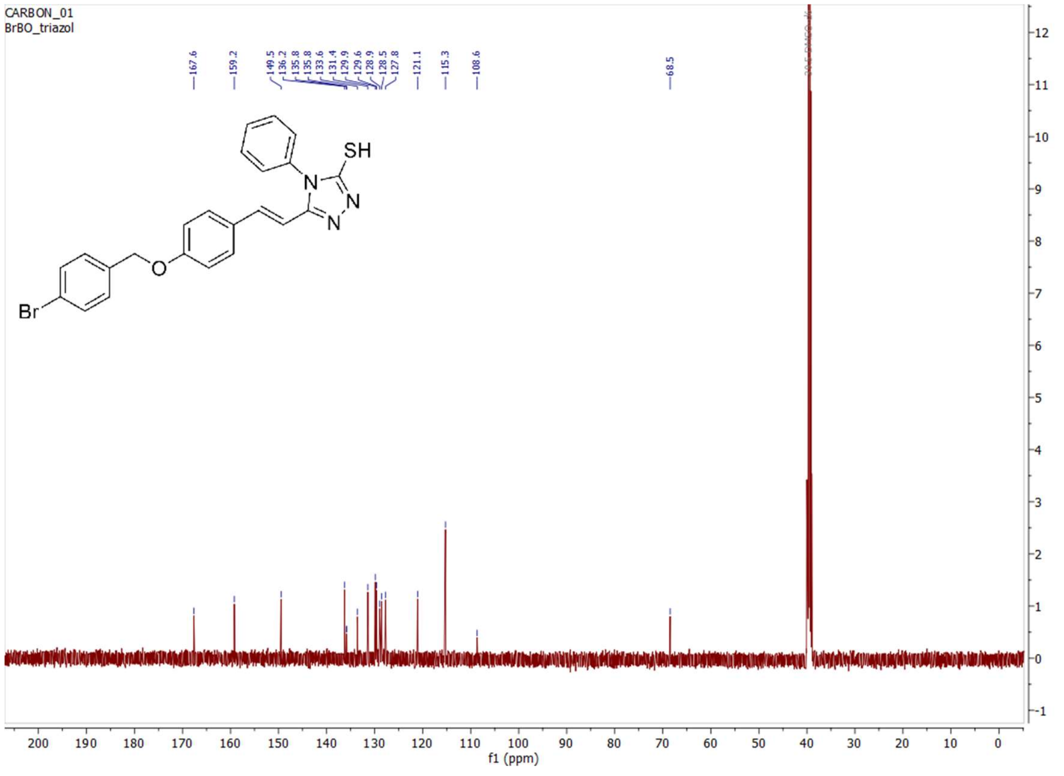

**Dimitra_Cl** corresponds to compound **5b**

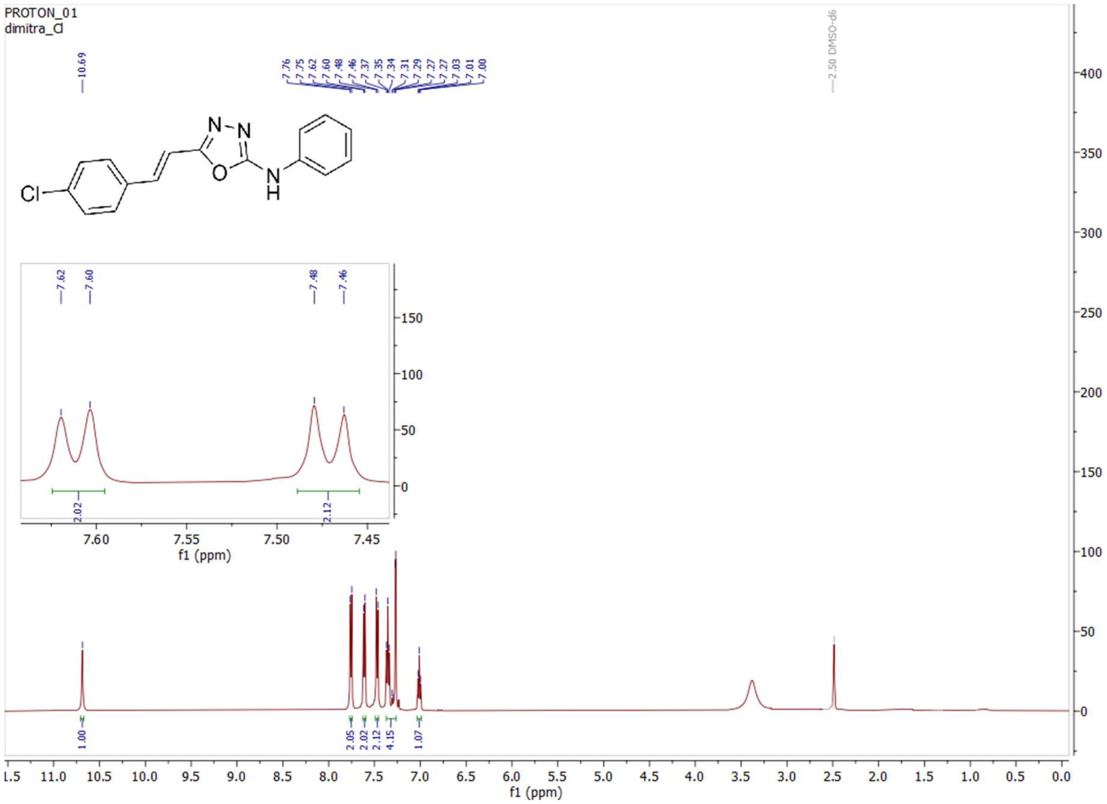


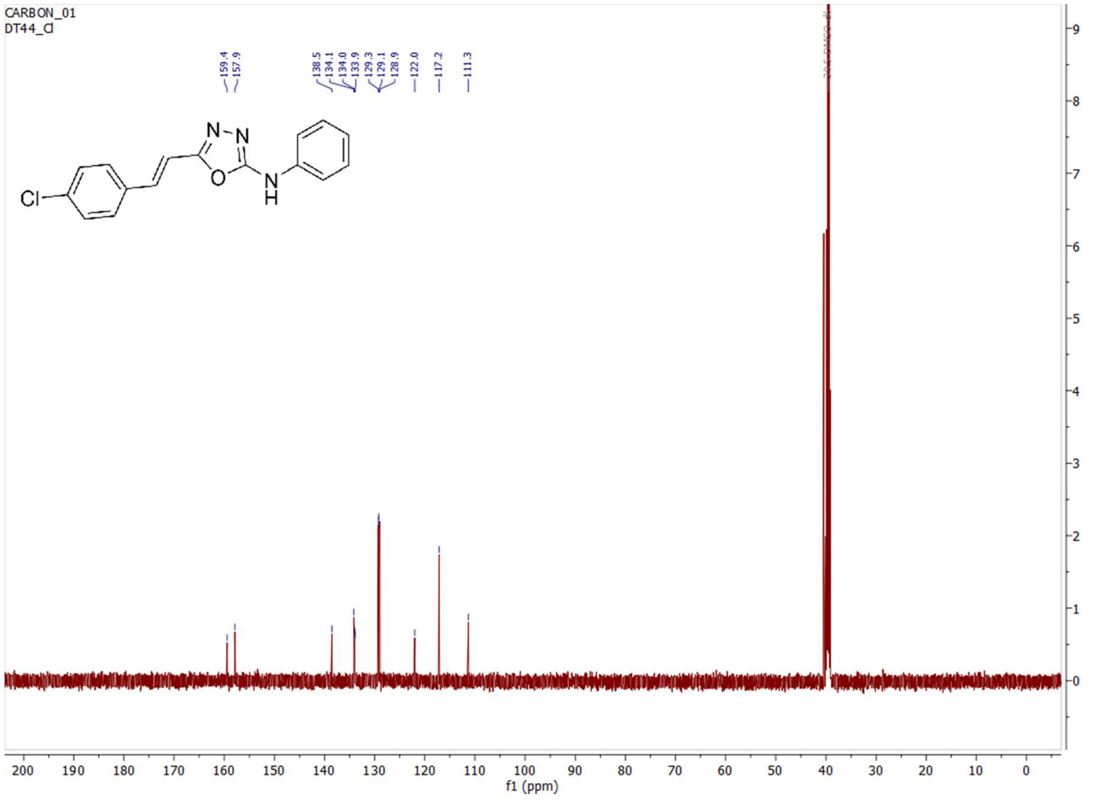

**Dimitra_F** corresponds to compound **5c**

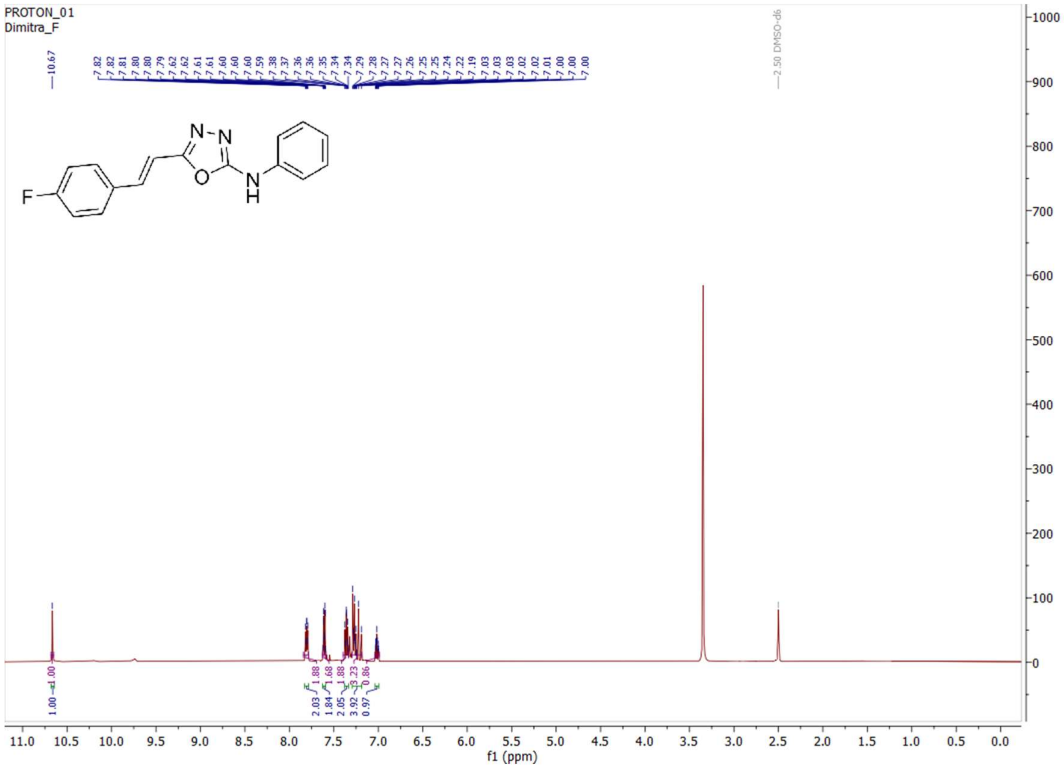

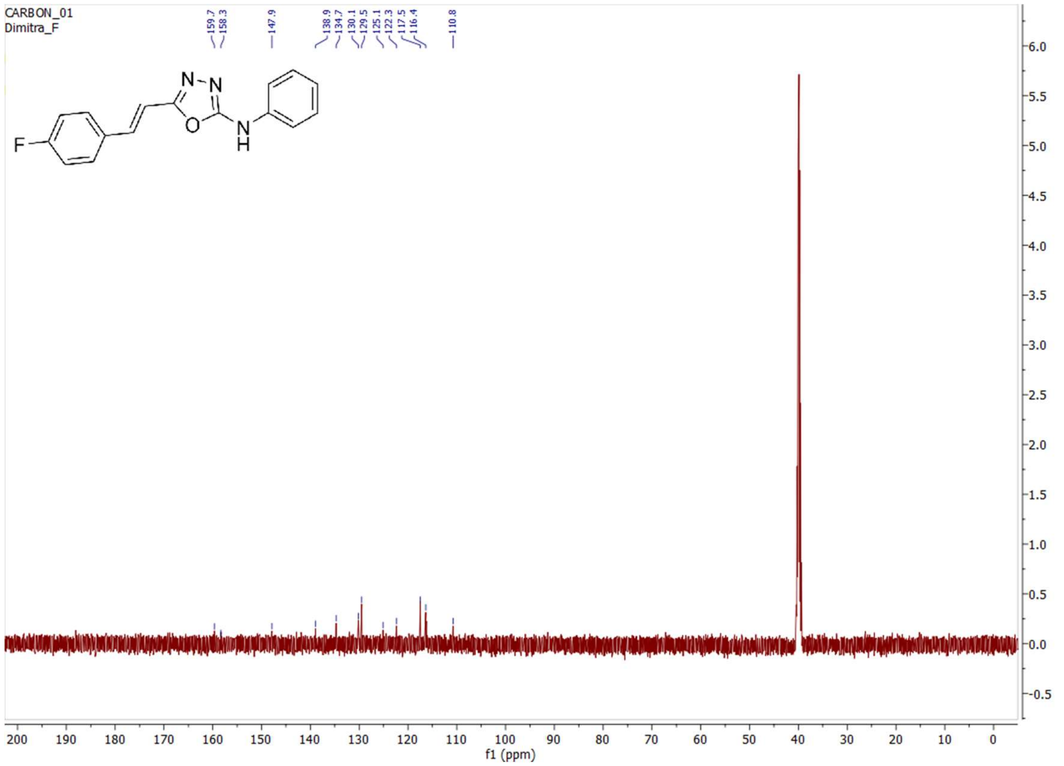

**pF_PITC** corresponds to compound **3c**

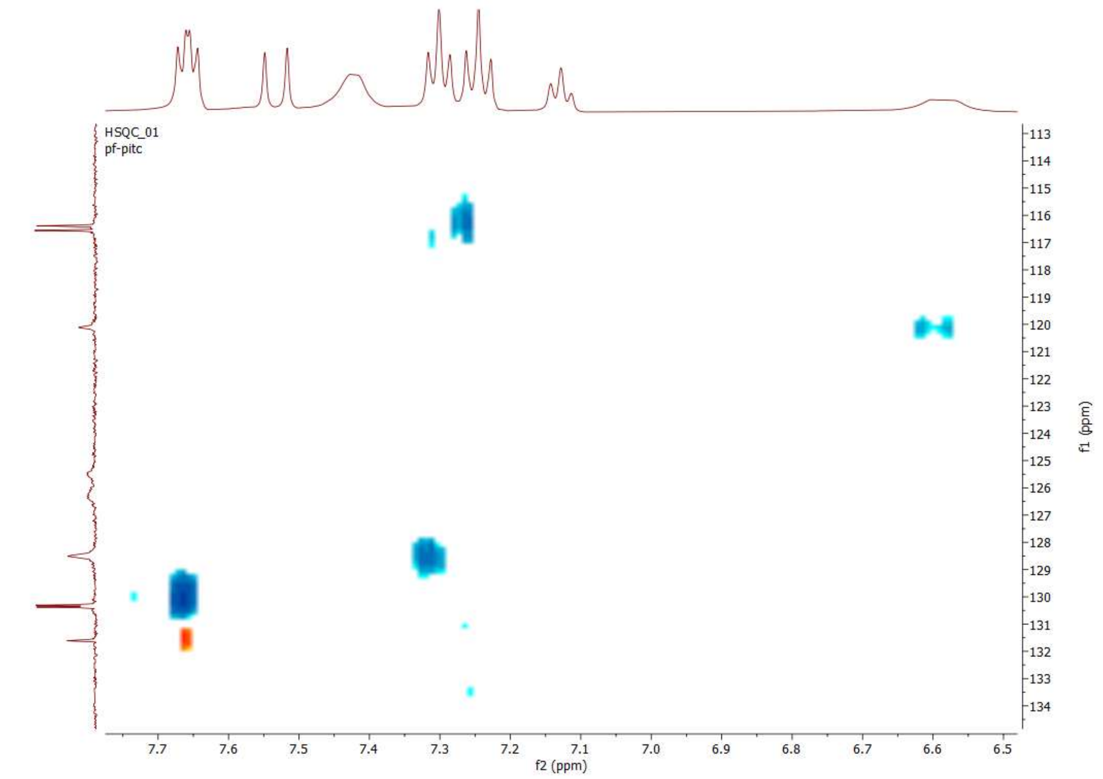


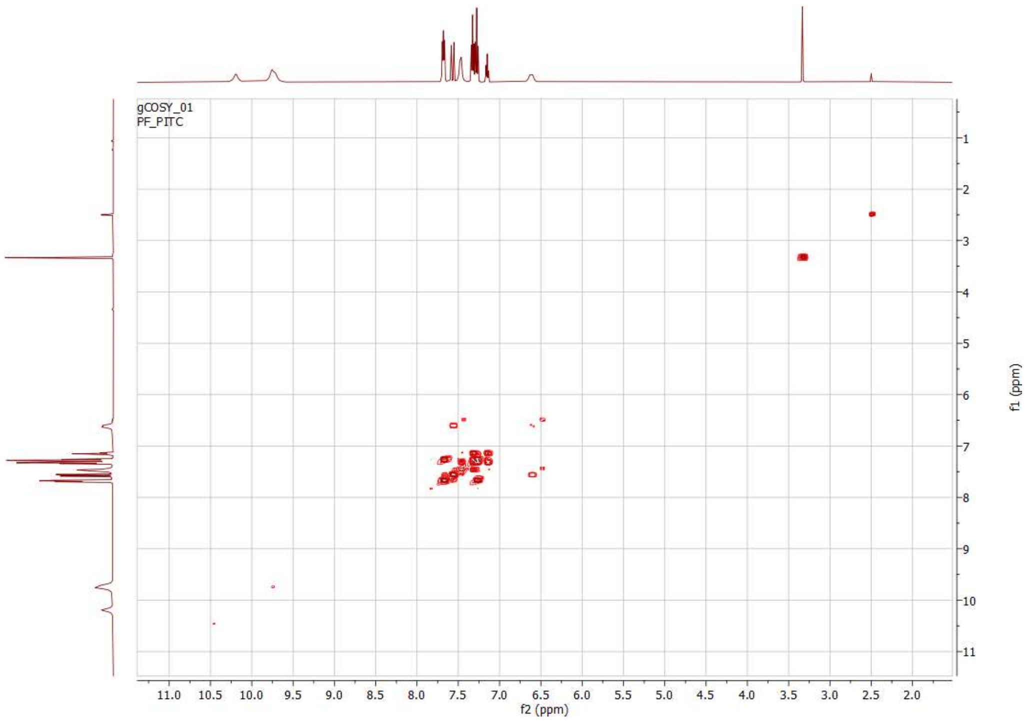

**TK25** corresponds to compound **4a**

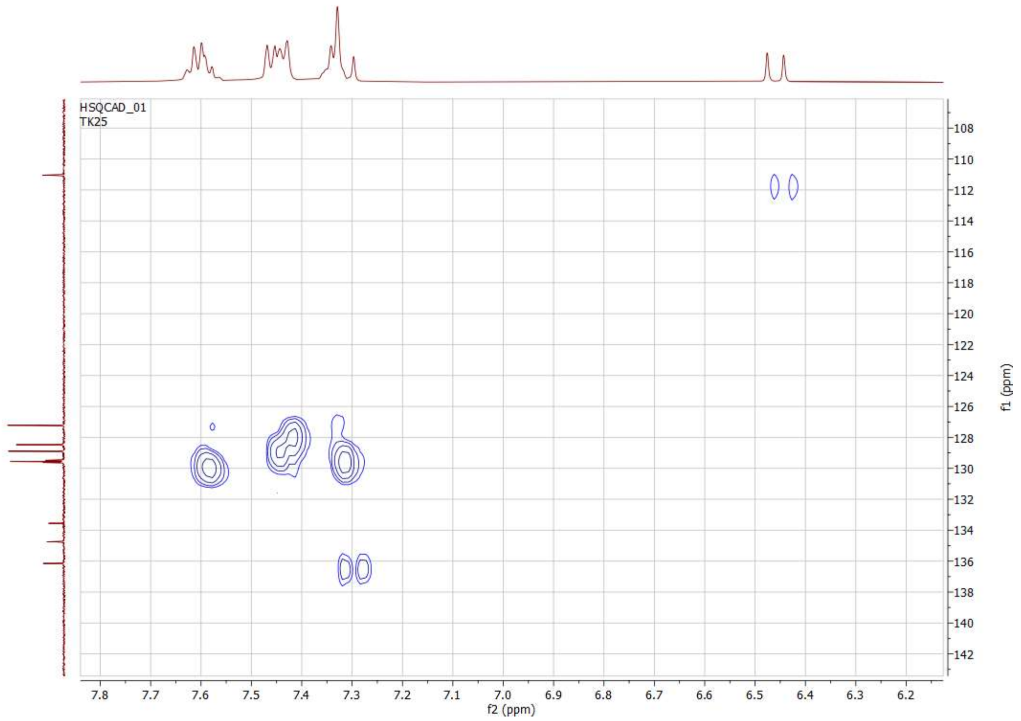


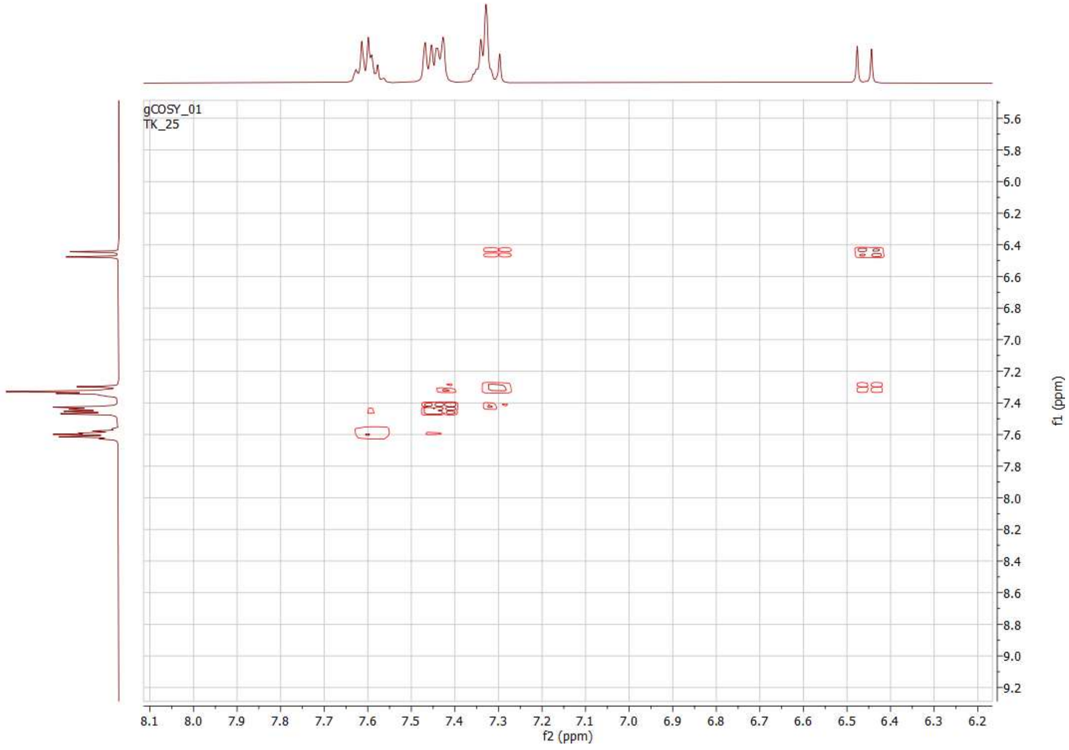

**Table S2.** Molecular docking studies of the synthesized compounds.

| Compd | Affinity (kcal/mol) | Hydrophobic interactions | Hydrogen bonds |
|---|---|---|---|
| **3a** | -7.8 | Val126, Val520, Lys526, Trp772 | Tyr525, Tyr529, Asp768 |
| **3b** | -8.1 | Val126, Val520, Tyr525, Lys526, Trp772 | Tyr525, Thr529, Thr529 |
| **3c** | -8 | Val126, Val520, Tyr525, Lys526, Trp772 | Tyr525, Thr529, Asp768 |
| **3d** | -8.3 | Val250, Tyr252, Lys526, Pro530, Trp772 | Thr529, Asp768 |
| **3e** | -8.2 | Leu246, Val520, Tyr525, Pro530, Arg533 | Val520, Trp772 |
| **3f** | -9 | Val520, Tyr525, Lys526, Pro530, Trp772 | Phe144, Tyr525, Thr529, Asp768 |
| **3g** | -9.9 | Val126, Phe143, Leu246, Val520, Tyr525, Lys526, Arg533, Trp772 | Arg767, Asp768 |
| **4a** | -8.1 | Val126, Phe143, Val520, Lys526, Trp772 | Tyr525 |
| **4b** | -7.8 | Val126, Val520, Lys526, Trp772 | - |
| **4c** | -8.1 | Val126, Val520, Tyr525, Lys526, Trp772 | - |
| **4d** | -7.7 | Val126, Asp243, Val520, Tyr525, Lys526 | Phe144 |
| **4e** | -8.5 | Val126, Val520, Lys526, Tyr532, Trp772 | - |
| **4f** | -9.2 | Val126, Asp243, Val520, Tyr525, Lys526 | Phe144, Arg182 |
| **4g** | -9.2 | Val126, Tyr525, Lys526, Arg533, Trp772 | - |
| **5a** | -8.7 | Val126, Val520, Pro530, Arg533, Val762, Asp768 | Thr529 |
| **5b** | -8.8 | Phe143, Val520, Pro530, Arg533 | Cys147, Tyr525 |
| **5c** | -8.6 | Val520, Arg533 | Cys127, Tyr525 |
| **6a** | -8.6 | Val126, Val520, Pro530, Arg,533, Val762, Asp768 | Asn128 |

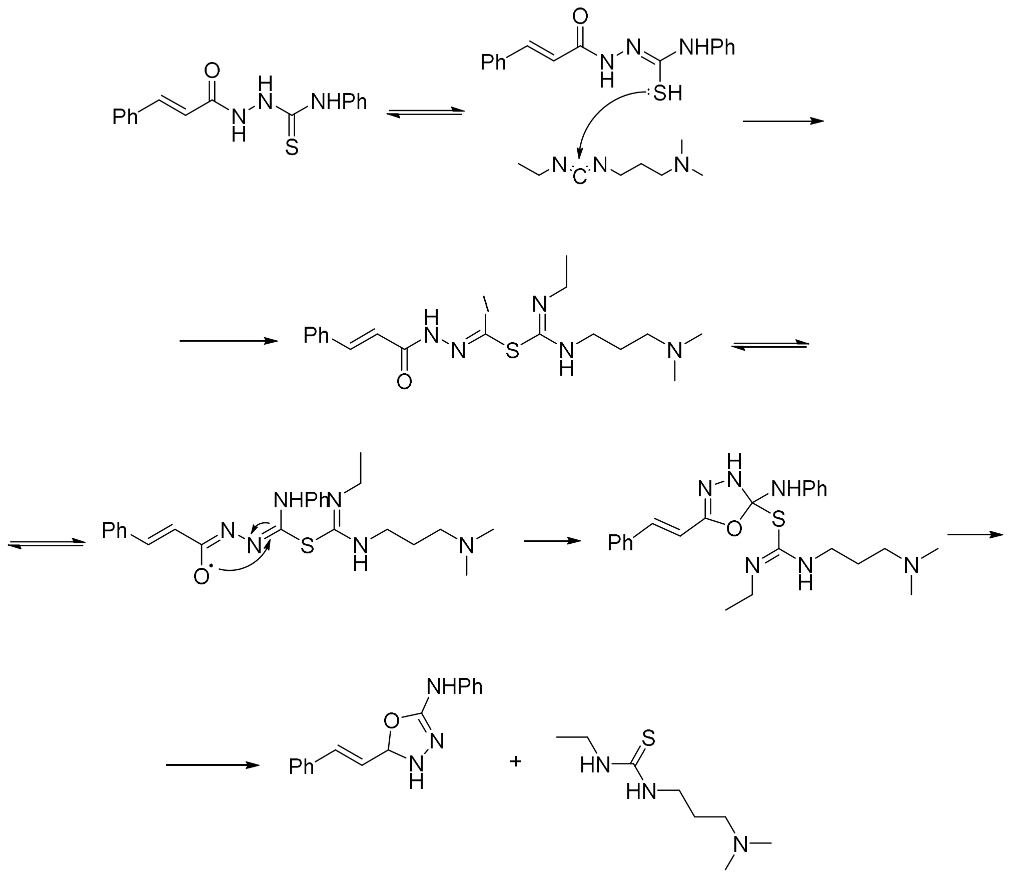


**Scheme S1.** Plausible reaction mechanism for the formation of oxadiazoles.
